# Comparative associations of six predefined dietary patterns with site-specific cancer risk in the UK Biobank

**DOI:** 10.3389/fnut.2026.1976233

**Published:** 2026-10-08

**Authors:** Long Gao, Pei Feng, Guofeng Shi, Yu Li, Bochen Ma, Chenqi Zhang, Yu Chen, Shaonan Li, Jingxi Hu, Liqiang Wang, Xiaoting Yan, Pengliang Shen, XiaoMing Cao, Bo Wu

**Affiliations:** 1 Department of Urology, First Hospital of Shanxi Medical University, Taiyuan, China; 2 School of Public Health, Shanxi Medical University, Taiyuan, China

**Keywords:** cancer risk, dietary patterns, UK Biobank, colorectal cancer, kidney cancer, lung cancer, polygenic risk score

## Abstract

**Background:**

Previous studies on diet and cancer have mainly examined individual dietary indices in relation to specific cancer outcomes, limiting direct comparison across dietary patterns. A unified population with consistent adjustment and standardized dietary-index scales is needed to explore whether different dietary patterns show different statistical associations with each specific cancer outcome.

**Methods:**

We included 118,056 cancer-free UK Biobank participants with at least two valid 24-h dietary assessments. Six predefined dietary indices were evaluated: DRLCD, aMED, AHEI-2010, DASH, CDAI, and E-DII. All indices were standardized to enable direct comparison. Progressively adjusted Cox regression models and 1-, 3-, and 5-year lagged sensitivity analyses were used as the primary analysis to identify the most stable diet–cancer outcome pairs. In the exploratory analysis, after excluding potential non-linear associations, standardized dietary indices were compared for colorectal, kidney, and lung cancers in the overall population and high genetic risk group to compare the direction and magnitude of associations across dietary patterns for each cancer outcome.

**Results:**

Eleven robust diet–cancer associations were identified. AHEI-2010 and DASH were inversely associated with colorectal cancer; AHEI-2010, aMED, CDAI, DASH, and reversed E-DII were inversely associated with kidney cancer; AHEI-2010, aMED, and reversed E-DII were inversely associated with lung cancer; and reversed E-DII was associated with lower overall cancer risk. DRLCD showed no robust association. In cross-index comparisons, the lowest HRs in the overall population were for DASH (colorectal cancer), reversed E-DII (kidney cancer), and AHEI-2010 (lung cancer); in the higher PRS group, they were for DASH (colorectal cancer), AHEI-2010 (kidney cancer), and reversed E-DII (lung cancer). However, bootstrap pairwise comparisons were not statistically significant, suggesting directional trends, not evidence of superiority.

**Conclusion:**

We identified significant and stable diet–cancer risk associations and observed differences in the direction or magnitude of associations across dietary indices. Because bootstrap pairwise comparisons were not statistically significant, these findings should be interpreted as directional trends and hypothesis-generating, not as evidence of a definitively optimal dietary strategy or dietary superiority.

## 1 Introduction

Cancer remains a major global public health challenge, with nearly 20 million new cases and 9.7 million cancer-related deaths worldwide in 2022 (1). As a key modifiable exposure, diet plays an important role in cancer prevention. Evidence-based dietary improvement may help reduce cancer risk and support population-level prevention strategies (2, 3). Therefore, clarifying the association between dietary patterns and cancer outcomes is essential for developing potential nutrition-based interventions.

In nutritional epidemiology, research has shifted from single nutrients or foods to dietary patterns that better reflect habitual intake. Compared with individual dietary components, dietary pattern approaches capture the synergistic and substitutive effects of foods, offering greater holistic relevance and interpretability for chronic disease and cancer prevention research (4). Accordingly, predefined dietary indices have been widely used in cancer epidemiology, including the Dietary Reduced-Carbohydrate Diet Score (DRLCD) (5), the Alternate Mediterranean Diet Score (aMED) (6), the Alternative Healthy Eating Index 2010 (AHEI-2010) (7), the Dietary Approaches to Stop Hypertension score (DASH) (8), the Combined Dietary Antioxidant Index (CDAI) (9), and the Energy-adjusted Dietary Inflammatory Index (E-DII) (10). Accumulating evidence suggests that healthier dietary patterns, such as aMED, AHEI-2010, and DASH, are generally associated with lower cancer risk, whereas more pro-inflammatory diets, reflected by higher DII or E-DII scores, have been linked to increased risks of several cancers (11–14).

Beyond diet, genetic susceptibility is a fundamental determinant of cancer risk. Polygenic risk scores (PRS), which integrate multilocus genetic variation, are increasingly used to quantify inherited cancer predisposition and identify high-risk individuals. Dietary factors may interact with or add to genetic predisposition, suggesting that unhealthy dietary patterns may be associated with higher cancer risk among genetically susceptible individuals (7). Therefore, genetic risk should be considered as an important stratification factor when evaluating whether the benefits of healthier diets differ by inherited susceptibility.

However, previous studies have often examined dietary indices separately or focused on specific cancer types, limiting direct comparisons across dietary patterns. It remains unclear whether different dietary patterns show similar, stronger, or more stable associations with the same cancer outcome, particularly across genetically defined risk subgroups. Evidence is especially limited among individuals at high genetic risk, for whom identifying dietary patterns that show inverse associations may have greater relevance for risk stratification. Therefore, a unified large-scale prospective cohort is needed to systematically compare the direction and magnitude of associations between diverse dietary patterns and site-specific cancer risks in the overall population and across genetic risk strata.

Therefore, in this prospective UK Biobank (UKB) cohort, we aimed to achieve three objectives. First, we sought to identify dietary pattern index–cancer outcome pairs that were significantly associated with the risk of site-specific cancers and consistently remained robust in lagged sensitivity analyses. Second, we examined whether these associations persisted across different levels of genetic risk and further assessed potential interactions between dietary patterns and genetic susceptibility. Third, we compared the relative magnitude of associations across different dietary patterns for specific cancer outcomes, with particular attention to whether similar patterns were observed among individuals in the higher PRS group.

## 2 Method

### 2.1 Study design and study population

This study was a prospective cohort analysis based on the UKB. Dietary intake was assessed using the Oxford WebQ, a web-based 24-h dietary recall instrument. The initial assessment was conducted near baseline, followed by repeated assessments over time. This questionnaire has been developed and validated in UKB and other large prospective studies (15). To improve the stability and representativeness of dietary exposure assessment, we included only participants who completed at least two valid 24-h dietary recalls using the updated Oxford WebQ nutrient calculation (16) and used the mean of repeated measurements to reflect habitual dietary intake. Individuals with overall cancer diagnosis at baseline were excluded. Repeated 24-h recalls were collected at up to five post-baseline time points (Table 1). Although averaging repeated assessments improves estimation of habitual intake, it may not fully capture long-term dietary changes during follow-up.

**Table 1:** Baseline characteristics of the total population.

Variables	Total (*N* = 118,056)	Without any cancer (*N* = 96,920)	With any cancer (*N* = 21,136)	Statistics	*p*-value
Follow-up Time (years)	13.20 (12.50, 14.00)	13.40 (12.80, 14.10)	7.40 (4.10, 10.20)	*Z* = 213.183	**<0.001**
Diet recall times				*χ*^2^ = 5.317	0.15
2 times	44,851 (38)	36,893 (38.1)	7,958 (37.7)		
3 times	39,502 (33.5)	32,352 (33.4)	7,150 (33.8)		
4 times	28,358 (24)	23,238 (24)	5,120 (24.2)		
5 times	5,345 (4.5)	4,437 (4.6)	908 (4.3)		
Age	57.00 (50.00, 62.00)	56.00 (49.00, 62.00)	61.00 (55.00, 64.00)	*Z* = 68.938	**<0.001**
BMI	26.02 (23.57, 29.05)	25.96 (23.51, 29.01)	26.30 (23.87, 29.25)	*Z* = 10.391	**<0.001**
TDI	−2.35 (−3.75, 0.01)	−2.30 (−3.73, 0.06)	−2.53 (−3.84, −0.32)	*Z* = −10.276	**<0.001**
Total MET-min/week	1704.00 (815.00, 3213.00)	1702.50 (817.00, 3213.00)	1706.00 (813.00, 3211.25)	*Z* = 0.033	0.974
Sleep Duration (h/day)	7.00 (7.00, 8.00)	7.00 (7.00, 8.00)	7.00 (7.00, 8.00)	*Z* = 9.941	**<0.001**
Sedentary Duration (h/day)	4.50 (3.00, 6.00)	4.50 (3.00, 6.00)	4.50 (3.00, 6.00)	*Z* = 12.185	**<0.001**
DTE (kJ /day)	8430.83 (7179.00, 9861.68)	8406.89 (7157.44, 9839.76)	8540.41 (7283.82, 9968.06)	*Z* = 8.141	**<0.001**
Sex				*χ*^2^ = 552.817	**<0.001**
Female	65,459 (55.4)	55,279 (57.0)	10,180 (48.2)		
Male	52,597 (44.6)	41,641 (43.0)	10,956 (51.8)		
Race				*χ*^2^ = 158.388	**<0.001**
White	113,829 (96.5)	93,143 (96.1)	20,686 (97.9)		
Others	4,187 (3.5)	3,744 (3.9)	443 (2.1)		
Hypertension at Baseline				*χ*^2^ = 432.136	**<0.001**
Non	90,473 (76.6)	75,434 (77.8)	15,039 (71.2)		
Yes	27,583 (23.4)	21,486 (22.2)	6,097 (28.8)		
Diabetes at Baseline				*χ*^2^ = 41.529	**<0.001**
Non	113,557 (96.2)	93,389 (96.4)	20,168 (95.4)		
Yes	4,499 (3.8)	3,531 (3.6)	968 (4.6)		
CVD at Baseline				*χ*^2^ = 162.210	**<0.001**
Non	112,414 (95.2)	92,646 (95.6)	19,768 (93.5)		
Yes	5,642 (4.8)	4,274 (4.4)	1,368 (6.5)		
Smoking				*χ*^2^ = 260.670	**<0.001**
Never	67,613 (57.4)	56,554 (58.5)	11,059 (52.4)		
Ever	41,842 (35.5)	33,425 (34.6)	8,417 (39.9)		
Current	8,345 (7.1)	6,734 (7.0)	1,611 (7.6)		
Drinking				*χ*^2^ = 156.330	**<0.001**
Rarely/never	17,702 (15.0)	14,724 (15.2)	2,978 (14.1)		
≥1 time/month	41,816 (35.4)	34,956 (36.1)	6,860 (32.5)		
≥3 times/week	58,464 (49.6)	47,176 (48.7)	11,288 (53.4)		
DRLCD	15.00 (12.00, 18.00)	15.00 (12.00, 18.00)	15.00 (12.00, 18.00)	*Z* = 3.240	**0.001**
aMED	4.00 (3.00, 6.00)	4.00 (3.00, 6.00)	4.00 (3.00, 6.00)	*Z* = 0.893	0.372
AHEI_2010	55.75 (46.49, 64.96)	55.69 (46.38, 64.88)	56.04 (46.95, 65.28)	*Z* = 4.198	**<0.001**
DASH	24.00 (21.00, 27.00)	24.00 (21.00, 27.00)	24.00 (21.00, 27.00)	*Z* = 8.028	**<0.001**
CDAI	−0.44 (−2.59, 2.03)	−0.46 (−2.61, 2.01)	−0.36 (−2.48, 2.12)	*Z* = 4.599	**<0.001**
E-DII	−0.04 (−0.17, 0.08)	−0.04 (−0.17, 0.09)	−0.04 (−0.18, 0.08)	*Z* = −3.668	**<0.001**

Continuous variables are presented as mean ± SD or median (Q1, Q3), as appropriate, and were compared using the independent-samples *t*-test, Welch’s *t*-test, or Mann–Whitney *U* test. Categorical variables are presented as *n* (%) and were compared using Pearson’s *χ*^2^ test, continuity-corrected *χ*^2^ test, or Fisher’s exact test, as appropriate. The Statistics column reports *t*, Welch’s *t*, *Z*, or *χ*^2^ values; Fisher’s exact test reports only the *p*-value. Bold *p*-values indicate statistical significance (*p* < 0.05). BMI, body mass index; TDI, Townsend deprivation index; DTE, daily total energy intake; Total MET-min/week, Total Metabolic Equivalent minutes per week; CVD, cardiovascular disease; DRLCD, dietary reduced-carbohydrate diet score; aMED, alternate Mediterranean diet score; AHEI-2010, alternative healthy eating index 2010; DASH, dietary approaches to stop hypertension score; CDAI, combined dietary antioxidant index; E-DII, energy-adjusted dietary inflammatory index.

### 2.2 Dietary assessment and construction of dietary pattern indices

Using food group and nutrient intake data from the Oxford WebQ, we constructed six predefined dietary indices: DRLCD, aMED, AHEI-2010, DASH, CDAI, and E-DII. Nutritionally, aMED and AHEI-2010 primarily reflect overall diet quality and adherence to healthy eating patterns; DASH captures dietary features related to blood pressure control and metabolic health; E-DII and CDAI represent the inflammatory and antioxidant potential of the diet, respectively; and DRLCD, based on the proportional contributions of carbohydrates, fat, and protein to total energy intake, was used to characterize adherence to a low-carbohydrate dietary pattern.

Given the substantial influence of total energy intake on dietary components and pattern scores, we applied energy standardization when constructing the dietary indices and additionally adjusted for total energy intake in subsequent multivariable models to minimize confounding from overall dietary intake.

#### 2.2.1 Dietary reduced-carbohydrate diet score (DRLCD)

The DRLCD was constructed from the energy contributions of carbohydrates, protein, and total fat. Each macronutrient was categorized into 11 groups and scored from 0 to 10. Carbohydrate intake was reverse-scored, whereas protein and fat intakes were positively scored. The three scores were summed to generate a total DRLCD score ranging from 0 to 30, with higher scores indicating greater adherence to a low-carbohydrate, high-protein, high-fat dietary pattern (17).

#### 2.2.2 Alternate Mediterranean diet score (aMED)

After energy density standardization (per 1,000 kcal), components were dichotomized at the population median. Participants received 1 point for above-median intakes of vegetables, legumes, fruits, nuts, whole grains, and fish, and for a higher monounsaturated-to-saturated fatty acid ratio; 1 point for below-median red and processed meat intake; and 1 point for alcohol intake of 5–25 g/day. The nine components were summed to generate a total aMED score ranging from 0 to 9 (18).

#### 2.2.3 Alternative healthy eating index 2010 (AHEI-2010)

Each component was scored linearly from 0 to 10. Beneficial components, including vegetables, fruits, whole grains, nuts and legumes, seafood, and polyunsaturated fatty acids, were positively scored, whereas sugar-sweetened beverages, red and processed meat, and sodium were reverse-scored. Sodium was classified by sex-specific deciles, and alcohol intake was scored using sex-specific criteria. The 10 components were summed to yield a total AHEI-2010 score ranging from 0 to 100 (19).

#### 2.2.4 Dietary approaches to stop hypertension score (DASH)

Fruit, vegetables, nuts and legumes, whole grains, low-fat dairy, sodium, red and processed meat, and sugar-sweetened beverages were energy-adjusted per 1,000 kcal, then scored from 1 to 5 according to sex-specific quintiles. Beneficial components were positively scored, whereas sodium, red and processed meat, and sugar-sweetened beverages were reverse-scored. The eight components were summed to derive a total DASH score ranging from 8 to 40 (20).

#### 2.2.5 Combined dietary antioxidant index (CDAI)

Six antioxidant nutrients—vitamin A, vitamin C, vitamin E, selenium, zinc, and total carotenoids—were selected for the CDAI. After calculating energy densities per 1,000 kcal, each component was standardized as a *Z*-score, and the standardized values were summed to generate the total CDAI score, with higher scores indicating greater antioxidant-related dietary intake. CDAI is a composite estimate of dietary intake and not a direct measure of systemic antioxidant capacity, oxidative stress, or tissue redox status (21).

#### 2.2.6 Energy-adjusted dietary inflammatory index (E-DII)

Using internationally standardized reference means and standard deviations, 27 dietary components were energy-adjusted (per 1,000 kcal) and then transformed by *Z*-standardization, percentile conversion, and centering. These values were multiplied by their predefined inflammatory effect scores and summed to generate the overall E-DII score, with higher scores indicating a more pro-inflammatory diet. The total score was calculated as a weighted sum of the available dietary components (22). E-DII reflects the predicted inflammatory potential of the diet rather than a direct measure of systemic inflammation. In this study, the E-DII was constructed using 27 components available in UKB rather than the original 45. Missing components are an inherent limitation of UKB dietary data and, more broadly, of constructing any dietary pattern from finite dietary assessment tools. Nevertheless, previous evidence suggests that reducing DII components to approximately 30 does not materially weaken predictive validity; simplified DIIs show acceptable agreement with the full version and remain associated with inflammatory markers (23). Thus, although some anti-inflammatory components may be omitted, the 27-component E-DII remains a pragmatic and defensible approach in UKB. Its findings may be broadly comparable in direction to those from more comprehensive versions, although absolute scores and effect sizes may differ and should be interpreted with caution.

Detailed information on the food and nutrient items used for each dietary index, the energy-standardization procedures, and the specific scoring criteria is provided in Supplementary Table S1.

### 2.3 Definition of cancer outcomes

A total of 16 cancer outcomes were included, comprising overall cancer and 15 site-specific malignancies: esophagogastric, colorectal, hepatobiliary, pancreatic, lung, skin, breast, uterine, cervical, ovarian, prostate, kidney, urothelial, lymphoid and hematopoietic cancers, and other cancers. Cancer diagnoses were ascertained from hospital inpatient records of first primary diagnoses coded by the International Classification of Diseases, Tenth Revision (ICD-10; UKB field 41,270), and cancer incidence data linked to the UK national cancer registries (UKB field 40,006). For each outcome, the endpoint was the first incident cancer event, and time-to-event analyses used pre-specified variables for the interval from baseline to first diagnosis. Follow-up was censored on 31 October 2022, with loss to follow-up and death treated as censoring events. Cancer outcomes were defined as the first incident cancer after baseline; cancer events before completion of repeated dietary assessments were not excluded. Detailed definitions and extraction procedures are provided in Supplementary Table S2.

### 2.4 Covariate adjustment and handling of missing data

Following conventional confounding control frameworks in nutritional epidemiology and cancer prevention research, three regression models were constructed with progressive covariate adjustment. Model 1 was unadjusted. Model 2 adjusted for baseline cancer-related characteristics and comorbidities, including age at recruitment, race, sex, baseline hypertension, baseline diabetes, and baseline cardiovascular and cerebrovascular diseases (CVD). Model 3 further adjusted for modifiable lifestyle and socioeconomic factors related to cancer incidence, including smoking status, alcohol consumption, daily total energy intake (DTE), body mass index (BMI), Townsend deprivation index (TDI), total metabolic equivalent task minutes per week (MET), sleep duration, and sedentary time (7, 24). Breast, uterine, ovarian, and prostate cancers were treated as sex-specific outcomes; therefore, sex was not included in the corresponding models. Detailed covariate extraction information is provided in Supplementary Table S2, and baseline variable distributions are shown in Table 1.

Missingness was first assessed across all variables, and variables with >20% missing values were excluded. Remaining missing data were handled using multiple imputation by chained equations (MICE). Categorical variables were imputed using logistic or polytomous regression, and continuous variables using a normal model. Fifty imputed datasets were generated, analyses were performed across all datasets, and regression estimates were pooled using Rubin’s rules to account for imputation uncertainty.

### 2.5 Event count assessment for model stability

To ensure stable regression estimates and avoid overfitting, we applied the events per variable (EPV) criterion of 10 to determine the minimum required outcome events for fully adjusted Model 3. Each continuous or binary covariate contributed one parameter, and each three-category covariate contributed two parameters. Model 3 included 7 continuous covariates, 5 binary covariates, 2 three-category covariates, and 1 continuous dietary index, yielding 17 parameters and a minimum requirement of 170 events. Cervical cancer had only 52 events (Supplementary Table S3), failed to meet this threshold, and was therefore excluded from subsequent analyses.

### 2.6 Standardization for comparability across dietary pattern indices

To enable direct comparison of association magnitudes between different dietary pattern indices and the same cancer outcome, all six indices were standardized before analysis. Each index was transformed into a z-score by subtracting the sample mean and dividing by the sample standard deviation. Thus, hazard ratios (HRs) from Cox models represent the change in cancer risk per 1-standard deviation (SD) increase in each dietary index, allowing comparisons under the same analytical framework. However, *z*-score standardization does not make the underlying dietary constructs biologically equivalent; cross-index comparisons should be interpreted as comparisons of statistical associations. Because higher E-DII scores indicate greater dietary inflammatory potential, E-DII was reverse-standardized so that higher values represented a less pro-inflammatory dietary pattern, consistent with the other health-oriented indices.

### 2.7 Primary analysis – identifying stable dietary patterns linked to incident cancer risk via cox regression and lagged sensitivity analysis

To preliminarily identify dietary patterns associated with 15 cancer outcomes, Cox proportional hazards models were fitted using the three progressively adjusted models described above. Dietary pattern–cancer combinations with statistically significant associations across all three models were defined as significant combinations. Results were reported as HRs with 95% confidence intervals (CIs).

Because cancers diagnosed shortly after baseline may reflect preclinical disease or disease-related dietary changes, leading to reverse causation, lagged sensitivity analyses were performed for the initially identified diet–cancer combinations (25). Based on Model 3, 1-, 3-, and 5-year lag windows were applied by excluding cancer events occurring within each period after baseline, and associations were re-evaluated to assess robustness.

Diet–cancer combinations that remained statistically significant across all three progressively adjusted Cox models and in the 1-, 3-, and 5-year lagged sensitivity analyses were considered robust and were subsequently carried forward to exploratory analyses.

### 2.8 Extended analysis – dose–response analysis with restricted cubic spline (RCS) and quartile categorical models

To assess potential non-linear dose–response relationships between dietary patterns and cancer risk, restricted cubic spline (RCS) models were applied to cancers with stable associations in preceding analyses, with each dietary index modeled separately. Dietary indices were standardized as z-scores, and four knots were used to estimate HRs and 95% CIs across each index distribution under Model 3 adjustment, while testing for non-linearity. A non-linearity *p*-value > 0.05 was interpreted as supporting a linear association.

Quartile-based categorical Cox models were also constructed for selected diet–cancer combinations to compare cancer risks across dietary adherence levels. Quartiles were defined using standardized dietary index values from Q1 to Q4. For all indices, Q1 was the reference group, representing the lowest and least healthy dietary level; for reversed E-DII, Q1 represented the most pro-inflammatory group. Cancer risks in Q2, Q3, and Q4 were compared with Q1 under Model 3 adjustment to assess whether higher dietary index levels were associated with progressively different risks.

### 2.9 Extended analysis – joint analysis of dietary patterns and genetic risk

For dietary pattern–cancer pairs with stable associations, joint analyses of dietary patterns and PRS were performed to evaluate risk stratification under genetic susceptibility. Publicly available SNP scoring files were obtained from the Polygenic Score (PGS) Catalog, and PRSs were calculated in the UKB-RAP using embedded PLINK in the Swiss Army Knife application (26), aligned to GRCh37 (hg19), standardized, and categorized into tertiles. The highest PRS tertile was defined as the higher PRS group; this is a study-defined category and does not represent a clinical high-risk classification. Dietary pattern scores were similarly categorized, retaining the lowest and highest tertiles for joint visualization. HRs and 95% CIs were estimated under Model 3, with low genetic risk plus a healthy dietary pattern as the reference. Detailed PRS construction and gene–dietary pattern interaction analyses are provided in Supplementary methods.

### 2.10 Extended analysis – cross-index comparison of dietary associations within the same cancer outcome

To compare the relative magnitude of associations between different dietary patterns and the same cancer outcome, cross-index comparison analyses were conducted for diet–cancer combinations with stable associations in the preceding analyses. These analyses were performed separately in the overall population and in the high PRS genetic risk group. For each cancer outcome, Model 3–adjusted Cox models were fitted separately for each standardized dietary index using a common complete-case sample.

Because Cox regression estimates effects on the log hazard scale, the relative strength of association was evaluated using the absolute value of the log HR, namely abs(logHR). Among dietary indices showing an inverse association with cancer risk, the index with HR < 1 and the largest abs(logHR) was selected as the index with the lowest observed HR. This selection does not indicate statistical superiority or clinical recommendation.

To further compare the selected index with other dietary indices for the same cancer outcome, bootstrap resampling was performed 1,000 times. In each bootstrap sample, Cox models were refitted and beta coefficients were estimated for each dietary index. Pairwise differences were calculated as Δβ = β_selected − β_comparator, and the corresponding ratio of HRs was calculated as exp.(Δβ). A ratio of HRs < 1 indicated a stronger inverse association for the selected index than for the comparator.

## 3 Results

### 3.1 Participant selection and analytic dataset

Among the initial sample, 501,936 participants underwent UKB baseline assessment. After excluding those without at least two valid Oxford WebQ assessments, 126,669 participants remained. A further 8,613 individuals with cancer diagnosed before or on the baseline date were excluded, leaving 118,056 participants in the final analysis. The participant selection process and baseline characteristics are shown in Figure 1 and Table 1, respectively. Cancer incidence during follow-up is summarized in Supplementary Table S3.

**Figure 1:**
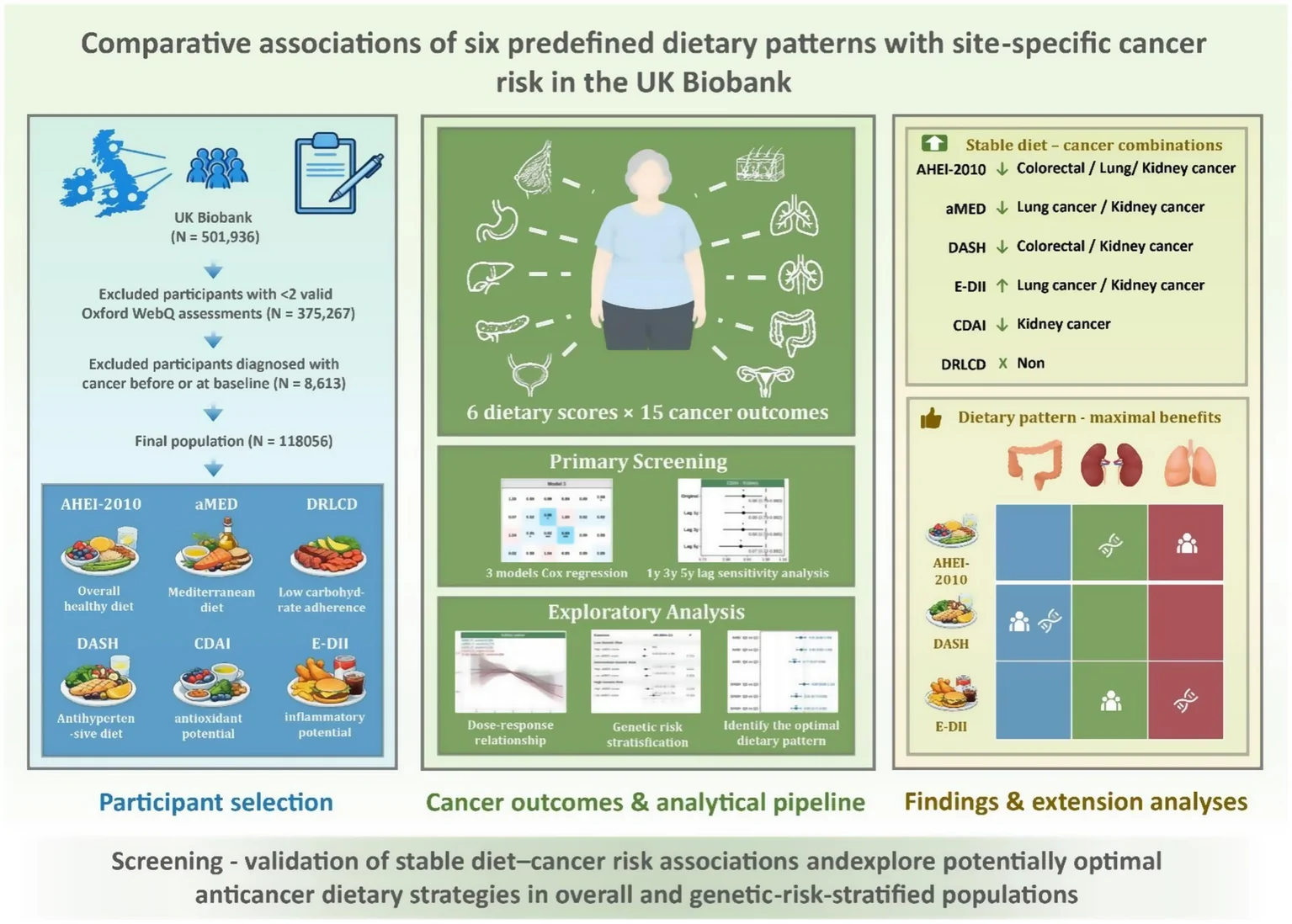
Study flowchart and overall analytical framework.

### 3.2 Primary screening and lagged sensitivity validation of diet–cancer associations

Progressively adjusted Cox models identified a subset of robust diet–cancer associations after full adjustment and lagged sensitivity analyses. Full Cox regression screening results are shown in Figure 2 and Supplementary Table S4, and lagged sensitivity analyses are shown in Figure 3 and Supplementary Table S5.

**Figure 2:**
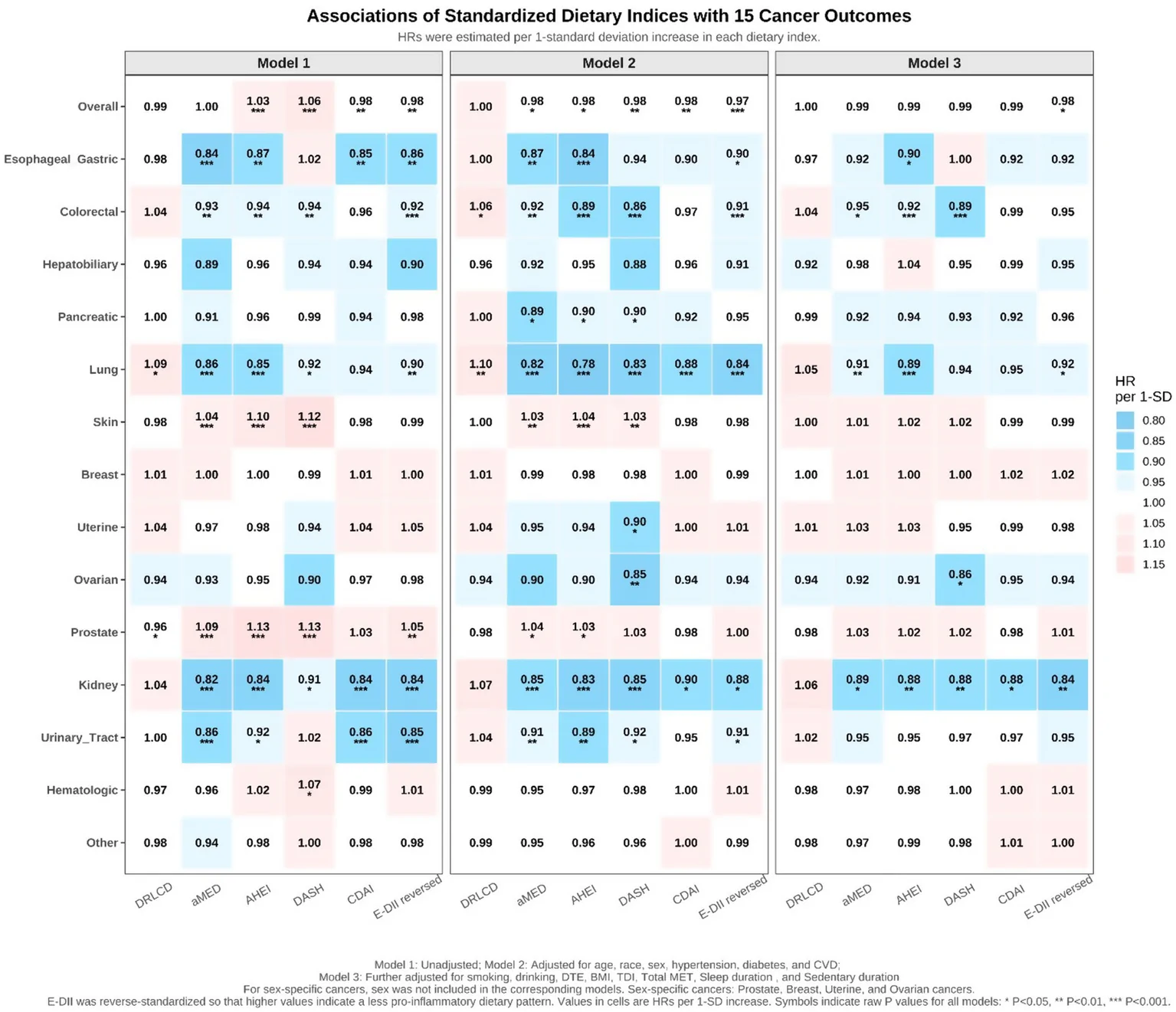
Primary cox regression screening of standardized dietary indices across 15 cancer outcomes.

**Figure 3:**
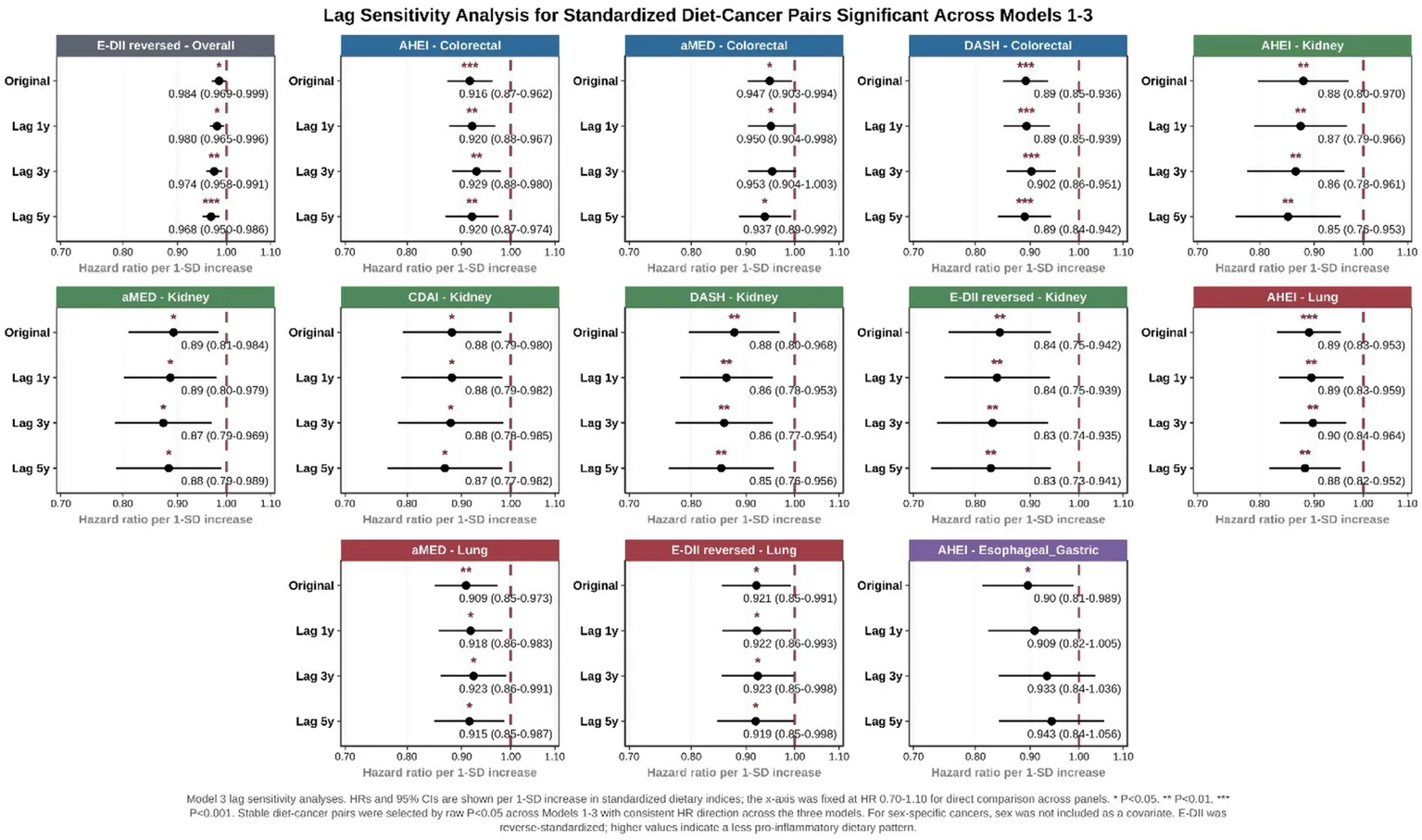
Lagged sensitivity analysis of diet–cancer combinations significant across progressive cox models.

DRLCD did not show significant associations with any cancer outcome in Model 3. For example, no significant association was observed for overall cancer (HR = 0.998, 95% CI: 0.984–1.012; *p* = 0.779), colorectal cancer (HR = 1.037, 95% CI: 0.989–1.087; *p* = 0.137), or lung cancer (HR = 1.054, 95% CI: 0.987–1.126; *p* = 0.118). Therefore, DRLCD was not carried forward to subsequent analyses.

For aMED, significant inverse associations were observed in Model 3 for colorectal cancer, kidney cancer, and lung cancer. Each 1-SD increase in aMED was associated with lower risks of colorectal cancer (HR = 0.947, 95% CI: 0.903–0.994; *p* = 0.027), kidney cancer (HR = 0.892, 95% CI: 0.810–0.984; *p* = 0.022), and lung cancer (HR = 0.909, 95% CI: 0.849–0.973; *p* = 0.006). For example, aMED was not significantly associated with overall cancer after full adjustment (HR = 0.992, 95% CI: 0.978–1.006; *p* = 0.242). In lagged sensitivity analyses, the associations with kidney and lung cancer remained stable, whereas the colorectal cancer association became non-significant in the 3-year lag analysis (HR = 0.953, 95% CI: 0.904–1.003; *p* = 0.067), and was therefore not retained as robust.

For AHEI-2010, Model 3 identified inverse associations with colorectal cancer, esophagogastric cancer, kidney cancer, and lung cancer. The associations with colorectal cancer (HR = 0.916, 95% CI: 0.873–0.962; *p* < 0.001), kidney cancer (HR = 0.879, 95% CI: 0.797–0.970; *p* = 0.010), and lung cancer (HR = 0.890, 95% CI: 0.831–0.953; *p* < 0.001) remained significant after 1-, 3-, and 5-year lag exclusions. By contrast, although the AHEI-2010–esophagogastric cancer association was significant in the original Model 3 analysis (HR = 0.896, 95% CI: 0.811–0.989; *p* = 0.029), it became non-significant after lagging, for example in the 1-year lag analysis (HR = 0.909, 95% CI: 0.822–1.005; *p* = 0.062) and the 5-year lag analysis (HR = 0.943, 95% CI: 0.842–1.056; *p* = 0.307).

For DASH, significant inverse associations in Model 3 were observed for colorectal cancer, kidney cancer, and ovarian cancer. The associations with colorectal cancer (HR = 0.892, 95% CI: 0.849–0.936; *p* < 0.001) and kidney cancer (HR = 0.878, 95% CI: 0.796–0.968; *p* = 0.009) were consistent across Models 1–3 and remained stable in all lagged analyses. Although DASH was associated with ovarian cancer in Model 3 (HR = 0.861, 95% CI: 0.768–0.965; *p* = 0.010), this association did not meet the predefined stability framework and was not retained.

For CDAI, only kidney cancer remained significantly associated in Model 3. Each 1-SD increase in CDAI was associated with lower kidney cancer risk (HR = 0.881, 95% CI: 0.792–0.980; *p* = 0.019), and this association remained significant after lagged exclusions. For other outcomes, the associations were generally weak or null. For example, CDAI was not significantly associated with overall cancer, although the estimate showed a borderline inverse trend (HR = 0.986, 95% CI: 0.971–1.000; *p* = 0.058).

For reversed E-DII, where higher values indicate lower dietary inflammatory potential, Model 3 showed inverse associations with overall cancer (HR = 0.984, 95% CI: 0.969–0.999; *p* = 0.042), kidney cancer (HR = 0.843, 95% CI: 0.755–0.942; *p* = 0.003), and lung cancer (HR = 0.921, 95% CI: 0.855–0.991; *p* = 0.029). These associations remained significant across lagged sensitivity analyses. In contrast, the association with colorectal cancer did not reach statistical significance after full adjustment (HR = 0.953, 95% CI: 0.904–1.005; *p* = 0.075).

Overall, 11 diet–cancer combinations were identified as stable and robust: reversed E-DII with overall cancer; AHEI-2010 and DASH with colorectal cancer; AHEI-2010, aMED, CDAI, DASH, and reversed E-DII with kidney cancer; and AHEI-2010, aMED, and reversed E-DII with lung cancer. These combinations were selected for further exploratory analyses.

### 3.3 Extended analyses for colorectal, kidney, and lung cancer

During PRS construction, colorectal cancer, kidney cancer, and lung cancer were represented using PGS003739 (81 SNPs, weighted by beta coefficients) (27), PGS004908 (107 SNPs, weighted by log odds ratios) (28), and PGP000148 (128 SNPs, weighted by log odds ratios) (29), respectively.

Among the robust cancer outcomes, colorectal cancer was associated with AHEI-2010 and DASH. RCS analyses did not suggest marked non-linearity, supporting the use of linear and categorical models. In quartile analyses, higher AHEI-2010 and DASH levels were associated with lower colorectal cancer risk. Compared with Q1, AHEI-2010 Q4 showed a significantly lower risk (HR = 0.774, 95% CI: 0.675–0.888; *p* < 0.001). For DASH, both Q3 and Q4 were significantly associated with lower risk, with HRs of 0.810 and 0.804, respectively. Dose–response and quartile-based analyses are presented in Supplementary Figures S2, S3, and Supplementary Table S6.

In PRS-stratified analyses, no significant interaction was observed between PRS and either AHEI-2010 or DASH for colorectal cancer. Nevertheless, in the high genetic risk group, both AHEI-2010 and DASH remained inversely associated with colorectal cancer risk, with identical HRs per 1-SD increase of 0.92. Joint analyses of dietary patterns and genetic risk are shown in Supplementary Figure S1 and Supplementary Table S7. In cross-index comparisons, DASH showed a slightly lower HR than AHEI-2010 in both the overall population and the high PRS group. However, bootstrap comparisons suggested only a directional trend, without statistical significance in the overall population (ratio HR = 0.974, 95% CI: 0.938–1.009; *p* = 0.122) or high PRS group (ratio HR = 0.991, 95% CI: 0.945–1.044; *p* = 0.756). These results should not be interpreted as evidence that DASH is superior to AHEI-2010. Cross-index comparisons in the overall population and PRS-stratified population are shown in Figures 4, 5 and Supplementary Tables S8, S9.

**Figure 4:**
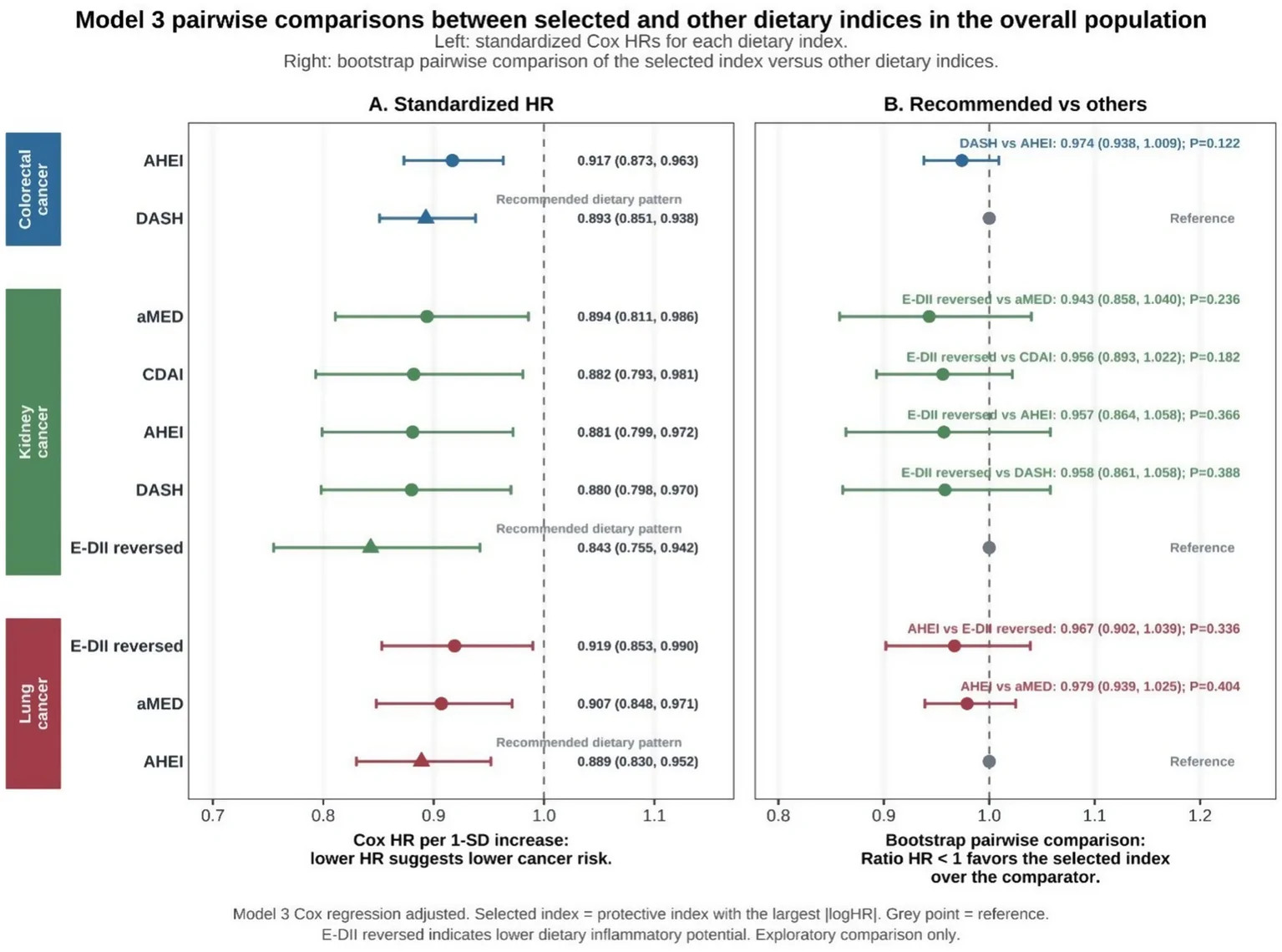
Model 3–adjusted pairwise comparison of selected and other dietary indices in the overall population.

**Figure 5:**
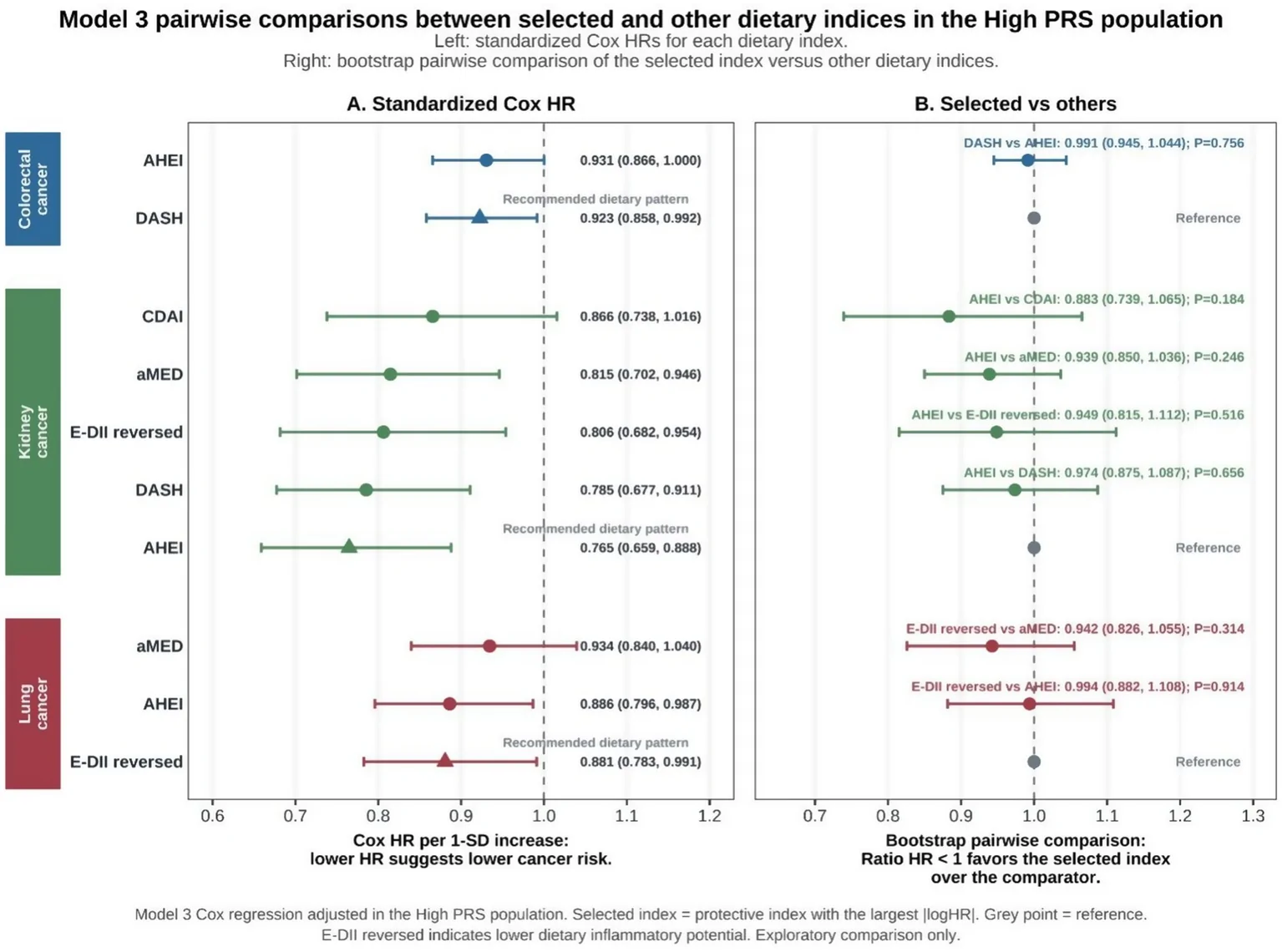
Model 3–adjusted pairwise comparison of selected and other dietary indices in the high PRS population.

Kidney cancer showed the broadest range of robust dietary associations. AHEI-2010, aMED, CDAI, DASH, and reversed E-DII were all inversely associated with kidney cancer risk. RCS analyses did not reveal obvious non-linearity. In quartile analyses, the highest quartile of each dietary index was significantly associated with lower kidney cancer risk compared with Q1, with Q4 versus Q1 HRs ranging from 0.637 for CDAI to 0.703 for reversed E-DII.

PRS-stratified analyses suggested potential modification by genetic susceptibility for several kidney cancer associations. Significant interactions were observed for AHEI-2010 (*P* interaction = 0.002), aMED (*P* interaction = 0.016), and DASH (*P* interaction = 0.013). In the high PRS group, AHEI-2010, aMED, DASH, and reversed E-DII remained inversely associated with kidney cancer risk, with HRs per 1-SD increase of 0.74, 0.81, 0.78, and 0.80, respectively. CDAI showed a similar direction but was not statistically significant in the high PRS stratum (HR = 0.88, 95% CI: 0.75–1.04; *p* = 0.142). In cross-index comparisons for kidney cancer, reversed E-DII showed the lowest HR in the overall population (HR = 0.843, 95% CI: 0.755–0.942; *p* = 0.003), whereas AHEI-2010 showed the lowest HR in the high PRS group (HR = 0.765, 95% CI: 0.659–0.888; *p* < 0.001). However, pairwise bootstrap comparisons showed only directional advantages. For example, in the overall population, reversed E-DII tended to show stronger inverse associations than aMED, CDAI, AHEI-2010, and DASH, but none of these comparisons reached statistical significance.

For lung cancer, AHEI-2010, aMED, and reversed E-DII were retained as robust dietary indices. RCS analyses did not indicate evident non-linearity. In quartile analyses, AHEI-2010 Q4 was significantly associated with lower lung cancer risk compared with Q1 (HR = 0.745, 95% CI: 0.614–0.904; *p* = 0.003). Reversed E-DII Q4 also showed a significant inverse association (HR = 0.802, 95% CI: 0.656–0.981; *p* = 0.032). For aMED, the association was significant in Q2 and Q3 but not in Q4, indicating a not consistently graded quartile pattern rather than a simple dose–response relationship.

No significant PRS interaction was observed for lung cancer. In the high PRS group, AHEI-2010 remained significantly associated with lower lung cancer risk when modeled continuously (HR = 0.88, 95% CI: 0.78–0.98; *p* = 0.022), whereas aMED (HR = 0.93, 95% CI: 0.84–1.04; *p* = 0.213) and reversed E-DII (HR = 0.90, 95% CI: 0.79–1.02; *p* = 0.098) did not reach statistical significance. In cross-index comparisons, AHEI-2010 showed the lowest HR in the overall population, while reversed E-DII showed a slightly lower HR in the high PRS group. These differences again represented directional trends rather than statistically significant pairwise differences.

Overall, no strong evidence of non-linearity was observed for colorectal, kidney, or lung cancer. In the overall population, the lowest HRs were observed for DASH in colorectal cancer, reversed E-DII in kidney cancer, and AHEI-2010 in lung cancer; in the high PRS group, the lowest HRs were observed for DASH in colorectal cancer, AHEI-2010 in kidney cancer, and reversed E-DII in lung cancer. However, these cross-index differences were not statistically significant and should be interpreted as directional trends.

## 4 Discussion

In this UKB prospective cohort study, we systematically compared six predefined dietary pattern indices with the risk of 15 incident cancer outcomes. After progressive Cox regression and 1-, 3-, and 5-year lagged sensitivity analyses, 11 robust diet–cancer associations were retained. AHEI-2010 and DASH were inversely associated with colorectal cancer; AHEI-2010, aMED, CDAI, DASH, and reversed E-DII were inversely associated with kidney cancer; AHEI-2010, aMED, and reversed E-DII were inversely associated with lung cancer; and reversed E-DII was also associated with lower overall cancer risk. DRLCD showed no robust association after full adjustment. In cross-index comparisons, the lowest HRs in the overall population were observed for DASH in colorectal cancer, reversed E-DII in kidney cancer, and AHEI-2010 in lung cancer. Among participants in the higher PRS group, the lowest HRs were observed for DASH in colorectal cancer, AHEI-2010 in kidney cancer, and reversed E-DII in lung cancer. However, bootstrap pairwise comparisons did not show statistically significant superiority of one dietary index over another; therefore, these findings should be interpreted as directional evidence of relative association magnitude rather than definitive ranking.

The main significance of this study is that it established a unified cross-index comparative framework. Previous nutritional epidemiology studies have usually focused on a “single dietary pattern–single cancer outcome” question, or evaluated different dietary indices in separate cohorts, populations, and statistical models. Such studies can suggest whether a specific dietary pattern is associated with a specific cancer, but cannot directly assess whether different dietary patterns show similar or directionally different associations with the same cancer outcome. To our knowledge, this is the first study to horizontally compare the associations with cancer risk of DRLCD, aMED, AHEI-2010, DASH, CDAI, and E-DII within the same large prospective cohort. Therefore, this study not only examined whether a given dietary pattern was associated with cancer risk, but also compared the direction and magnitude of associations across dietary patterns for the same cancer outcome.

To contextualize our findings, we manually selected representative previous studies in Supplementary Table S10 and compared their results with those of the present study. We did not conduct a formal systematic literature search. Overall, our results were partly consistent with previous evidence, but also showed several important differences. For aMED, previous meta-analytic evidence suggested inverse associations with several cancer sites, whereas in our study aMED was retained only for kidney and lung cancer after strict lagged validation, and its colorectal cancer association became unstable; this difference may reflect heterogeneity in Mediterranean diet scoring, population background, cancer definitions, and the more conservative nature of our repeated-diet, z-standardized, fully adjusted, and lag-validated framework (6). For DASH, previous meta-analyses supported a clear inverse association with colorectal cancer, which was highly consistent with our findings, whereas other cancer sites reported previously were not retained in our study; this may be because prior studies often used highest-versus-lowest comparisons or pooled risk estimates, while our study required stability across progressive models and lagged analyses (8, 30). For kidney cancer, our results were generally consistent with previous UKB evidence showing inverse associations for aMED, AHEI-2010, DASH, and lower dietary inflammatory potential, especially among genetically susceptible individuals; however, our study further extended this evidence by screening across 15 cancer outcomes and adding CDAI, reversed E-DII, and bootstrap cross-index comparison (7). For dietary inflammatory potential, previous dose–response meta-analysis reported positive associations of DII/E-DII with overall, colorectal, lung, and kidney cancers, whereas in our study reversed E-DII remained robust for overall, kidney, and lung cancer but not colorectal cancer; this discrepancy may result from lower statistical power within a single cohort, stricter confounder adjustment, and exclusion of early events through lagged analyses (10). For lung cancer, previous Multiethnic Cohort evidence supported inverse associations for multiple diet quality indices, whereas our robust lung cancer indices were AHEI-2010, aMED, and reversed E-DII; differences in smoking structure, smoking-related residual confounding, dietary assessment tools, and population composition may partially account for observed differences (31). For aMED, the quartile pattern was not consistently graded, and this association should not be interpreted as a simple dose–response relationship. Importantly, the inverse associations observed for AHEI-2010, aMED, and reversed E-DII with lung cancer should be interpreted with particular caution, because smoking is a major risk factor for lung cancer and may be associated with dietary behaviors; residual confounding by smoking cannot be fully excluded. For DRLCD, previous Asian cohort studies have suggested inconsistent associations between low-carbohydrate dietary patterns and cancer risk, potentially reflecting differences in population dietary background, macronutrient replacement patterns, overall energy structure, and long-term dietary habits (5, 32). In the present study, DRLCD showed no robust association after full adjustment and lagged sensitivity analyses, suggesting that a low-carbohydrate tendency alone may not consistently capture the complex dietary exposures relevant to cancer development. For CDAI, although previous studies reported inverse associations with colorectal or lung cancer, our study retained a stable association only for kidney cancer, indicating that antioxidant-related dietary features may have more site-specific rather than universal associations with cancer risk (9, 33).

The biological mechanisms underlying the observed associations between healthy dietary patterns and lower cancer risk are likely multifactorial and may partly overlap across shared nutritional components. These proposed mechanisms are potential and were not directly demonstrated in the current study. Indices such as AHEI-2010, aMED, and DASH are characterized by higher intakes of fruits, vegetables, whole grains, and nuts, which provide dietary fiber, polyphenols, vitamins, and other bioactive compounds. Dietary fiber can be fermented by the gut microbiota to produce short-chain fatty acids (SCFAs), including butyrate, which may contribute to anti-inflammatory and anti-proliferative effects within the colorectal epithelium (34, 35). In addition, the inverse association observed for reversed E-DII is consistent with the potential role of systemic inflammation in cancer development. Pro-inflammatory dietary patterns, often characterized by higher intake of processed meats and refined carbohydrates, may contribute to chronic low-grade inflammation and tumor-promoting microenvironments through pathways involving nuclear factor-kappa B (NF-κB) and inflammatory cytokines such as interleukin-6 (IL-6) and tumor necrosis factor-alpha (TNF-α) (36). For kidney cancer, the relatively broad associations observed across multiple dietary indices may also be partly related to antioxidant-related mechanisms. Antioxidant nutrients, as reflected by CDAI, may help counteract reactive oxygen species (ROS) accumulation and attenuate lipid peroxidation and oxidative DNA damage in renal tissues, although this mechanism remains inferential in the present observational setting (37, 38).

These indices are not independent. AHEI-2010, aMED, and DASH share higher intakes of plant foods, fiber-rich foods, and lower intakes of red/processed meat. CDAI and reversed E-DII also overlap with these patterns through antioxidant-rich and less inflammatory foods. Therefore, differences across indices may partly reflect score construction and measurement rather than clearly distinct dietary effects.

Several limitations should be acknowledged. First, residual confounding cannot be fully excluded, particularly for lung cancer, where smoking pack-years, occupational exposure, and second-hand smoke may not be completely captured. Second, although repeated Oxford WebQ assessments were used and the mean of repeated 24-h dietary recalls was calculated to better approximate habitual intake, short-term dietary assessments may still be affected by day-to-day variation, recall bias, seasonal differences, and dietary changes during follow-up, potentially leading to misclassification of long-term dietary patterns. Such misclassification may have attenuated the observed associations and influenced cross-index comparisons when differences in effect sizes were modest. Third, UKB participants in this study were predominantly White, with White participants accounting for 96.5% of the analytic cohort; therefore, the generalizability of our findings to non-White populations may be limited. Differences in dietary habits, socioeconomic background, cancer risk profiles, genetic ancestry, and PRS performance across populations should be considered when interpreting these results. Fourth, although z-score standardization enabled cross-index comparison, different dietary indices share overlapping food components, and bootstrap comparisons did not prove statistically significant superiority of one index over another. Medication and supplement use were not adjusted for. Although these factors may be associated with cancer risk and dietary behaviors, the available UKB data on medication and supplement use are incomplete and mainly self-reported; adjusting for them could introduce measurement error and over-adjustment. Fifth, Supplementary Table S10 was based on manually selected representative studies rather than a formal systematic literature search. Finally, given this is an observational study, no effect or causation can be established.

## 5 Conclusion

In conclusion, these findings preliminarily suggest that different cancer outcomes showed differences in the direction or magnitude of associations across dietary patterns. Among the dietary indices compared, a DASH-represented healthy dietary pattern had the lowest observed HR for colorectal cancer in both the overall population and individuals in the higher PRS group. For kidney cancer, reversed E-DII, representing a lower-inflammatory diet, had the lowest observed HR in the overall population, whereas AHEI-2010 had the lowest observed HR in individuals in the higher PRS group. For lung cancer, AHEI-2010 had the lowest observed HR in the overall population, whereas reversed E-DII had the lowest observed HR individuals in the higher PRS group. However, bootstrap pairwise comparisons were not statistically significant; therefore, these findings should be interpreted as directional trends rather than evidence that one dietary pattern is superior to another.

## Data Availability

Publicly available datasets were analyzed in this study. This data can be found: https://www.ukbiobank.ac.uk.

## Glossary

aMED: Alternate Mediterranean Diet Score
AHEI-2010: Alternative Healthy Eating Index 2010
BMI: Body mass index
CDAI: Combined Dietary Antioxidant Index
CI: confidence interval
CVD: cardiovascular and cerebrovascular disease
DASH: Dietary Approaches to Stop Hypertension
DRLCD: Dietary Reduced-Carbohydrate Diet Score
DTE: daily total energy intake
E-DII: Energy-adjusted Dietary Inflammatory Index
EPV: events per variable
FDR: False discovery rate
GRCh37: Genome Reference Consortium Human Build 37
HR: hazard ratio
ICD-10: International Classification of Diseases, Tenth Revision
IL-6: interleukin-6
MET: Metabolic Equivalent of Task
MICE: Multiple imputation by chained equations
NF-κB: nuclear factor-kappa B
PRS: Polygenic risk score
PGS: Polygenic Score
PLINK: Whole-genome association analysis toolset
ROS: reactive oxygen species
RCS: Restricted cubic spline
SNP: Single nucleotide polymorphism
SCFAs: short-chain fatty acids
SD: standard deviation
TDI: Townsend deprivation index
TNF-α: tumor necrosis factor-alpha
UKB: UK Biobank
UKB-RAP: UK Biobank Research Analysis Platform
