# Supplementary material for: Comparative associations of six predefined dietary patterns with site-specific cancer risk in the UK Biobank
Source: Front Nutr. 2026 Oct 8;13:1976233. doi: 10.3389/fnut.2026.1976233 (PMC13686994; doi:10.3389/fnut.2026.1976233)
Supplement: Supplementary file 1 [file Image_1.PDF]

PRS-Stratified Diet-Cancer Associations in Selected Pairs

Joint display by genetic risk and standardized dietary score; interaction tested using continuous PRS × continuous standardized diet score

A Colorectal cancer × AHEI

Interaction P = 0.121
High genetic risk: diet HR per 1-SD = 0.92 (0.86-0.99); P = 0.035

Exposure	Outcome	Case	Control	HR (95% CI)	P
**Low Genetic Risk**					
High AHEI score	Colorectal cancer	112	12936	Ref	
Low AHEI score	Colorectal cancer	139	12502	1.33 (1.04-1.72)	0.025
**Intermediate Genetic Risk**					
High AHEI score	Colorectal cancer	167	12643	1.51 (1.19-1.92)	<0.001
Low AHEI score	Colorectal cancer	195	12669	1.81 (1.43-2.29)	<0.001
**High Genetic Risk**					
High AHEI score	Colorectal cancer	262	12461	2.40 (1.92-2.99)	<0.001
Low AHEI score	Colorectal cancer	285	12791	2.63 (2.10-3.28)	<0.001

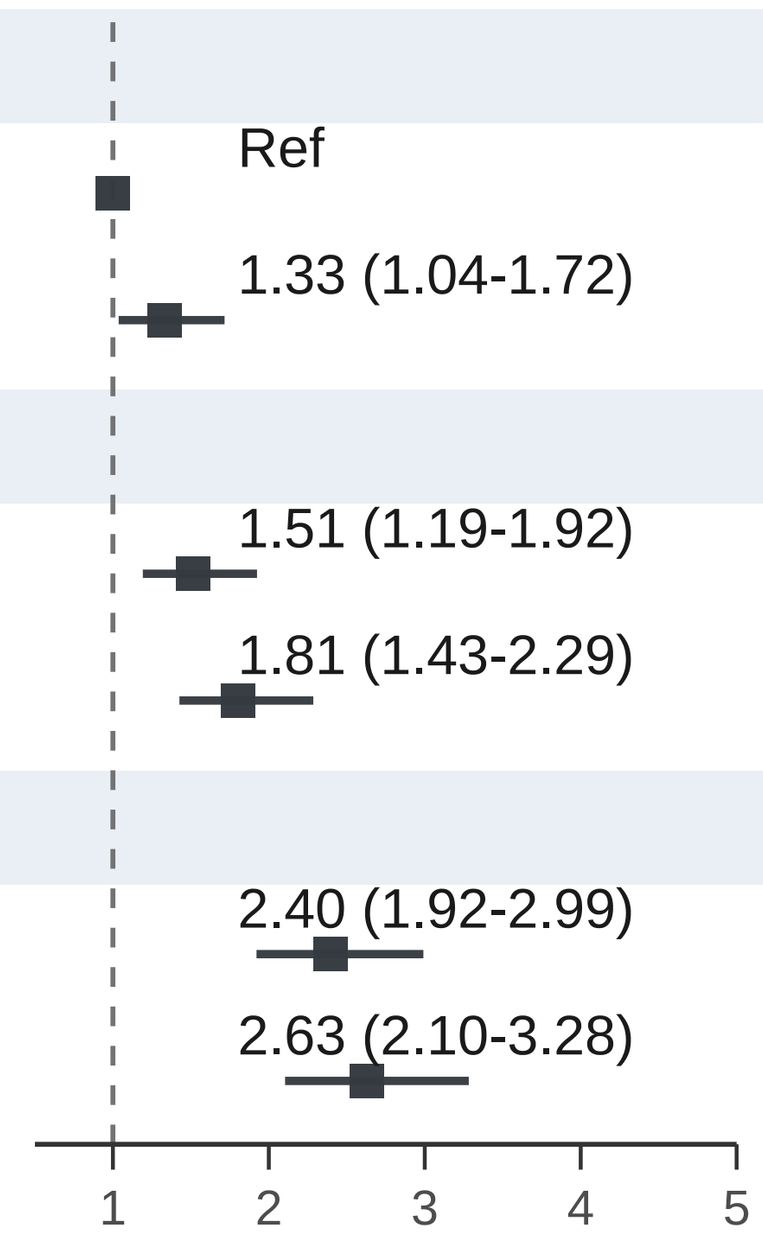

B Colorectal cancer × DASH

Interaction P = 0.183
High genetic risk: diet HR per 1-SD = 0.92 (0.86-0.99); P = 0.024

Exposure	Outcome	Case	Control	HR (95% CI)	P
**Low Genetic Risk**					
High DASH score	Colorectal cancer	127	14845	Ref	
Low DASH score	Colorectal cancer	165	14443	1.50 (1.18-1.89)	<0.001
**Intermediate Genetic Risk**					
High DASH score	Colorectal cancer	188	14645	1.50 (1.19-1.87)	<0.001
Low DASH score	Colorectal cancer	245	14535	2.14 (1.72-2.66)	<0.001
**High Genetic Risk**					
High DASH score	Colorectal cancer	314	14388	2.53 (2.06-3.11)	<0.001
Low DASH score	Colorectal cancer	333	14579	2.93 (2.38-3.60)	<0.001

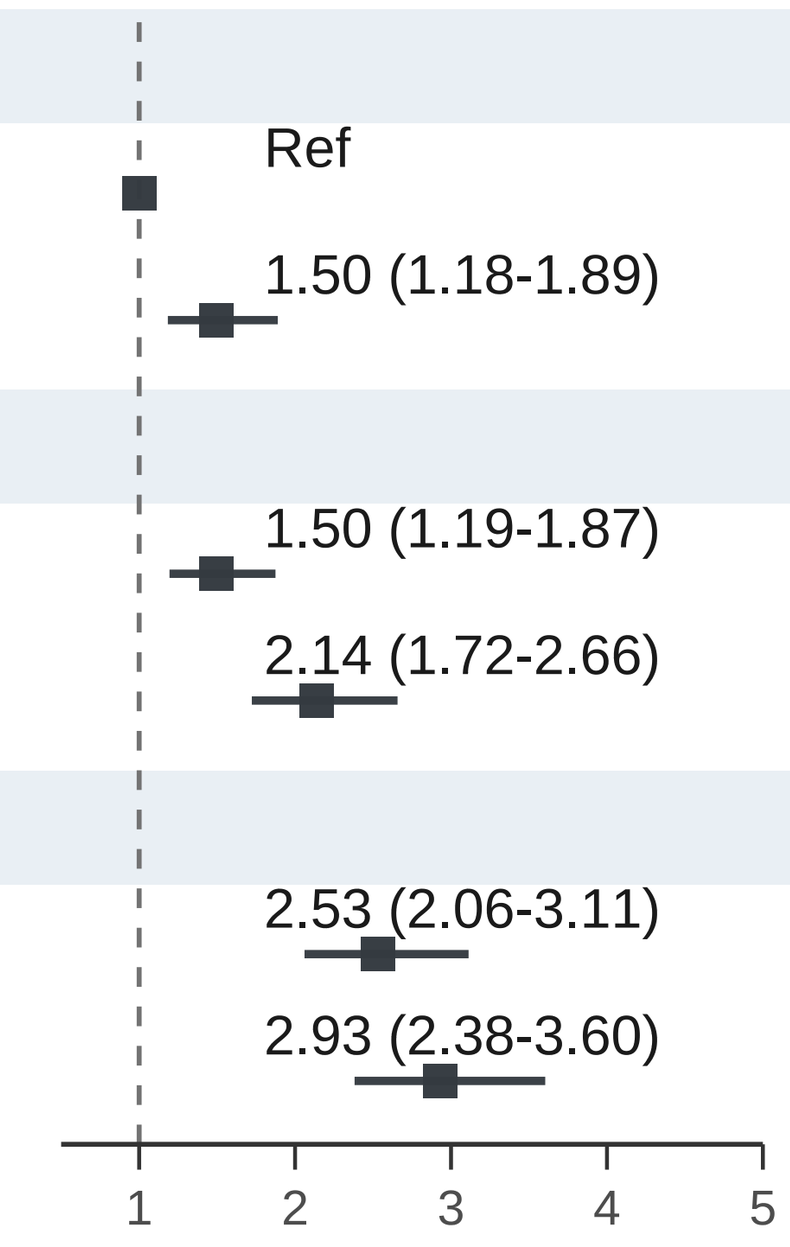

C Kidney cancer × AHEI

Interaction P = 0.002
High genetic risk: diet HR per 1-SD = 0.74 (0.63-0.87); P = <0.001

Exposure	Outcome	Case	Control	HR (95% CI)	P
**Low Genetic Risk**					
High AHEI score	Kidney cancer	27	12789	Ref	
Low AHEI score	Kidney cancer	36	12884	1.19 (0.72-1.98)	0.495
**Intermediate Genetic Risk**					
High AHEI score	Kidney cancer	41	12909	1.50 (0.92-2.44)	0.102
Low AHEI score	Kidney cancer	44	12839	1.41 (0.87-2.29)	0.164
**High Genetic Risk**					
High AHEI score	Kidney cancer	39	12776	1.42 (0.87-2.32)	0.161
Low AHEI score	Kidney cancer	87	12691	2.90 (1.87-4.49)	<0.001

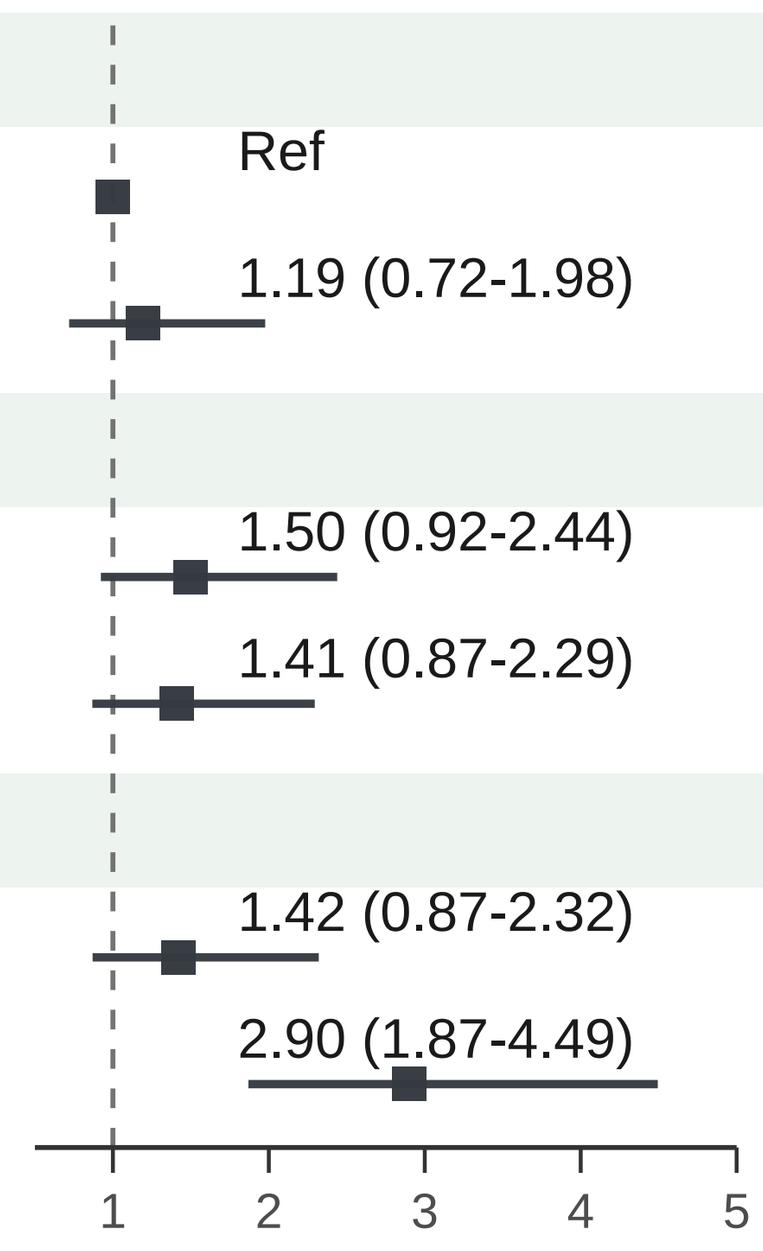

D Kidney cancer × aMED

Interaction P = 0.016
High genetic risk: diet HR per 1-SD = 0.81 (0.70-0.95); P = 0.007

Exposure	Outcome	Case	Control	HR (95% CI)	P
**Low Genetic Risk**					
High aMED score	Kidney cancer	49	18028	Ref	
Low aMED score	Kidney cancer	60	20444	0.93 (0.64-1.36)	0.723
**Intermediate Genetic Risk**					
High aMED score	Kidney cancer	56	18074	1.13 (0.77-1.66)	0.521
Low aMED score	Kidney cancer	78	20372	1.19 (0.83-1.71)	0.333
**High Genetic Risk**					
High aMED score	Kidney cancer	66	18080	1.33 (0.92-1.93)	0.129
Low aMED score	Kidney cancer	128	20306	1.97 (1.41-2.75)	<0.001

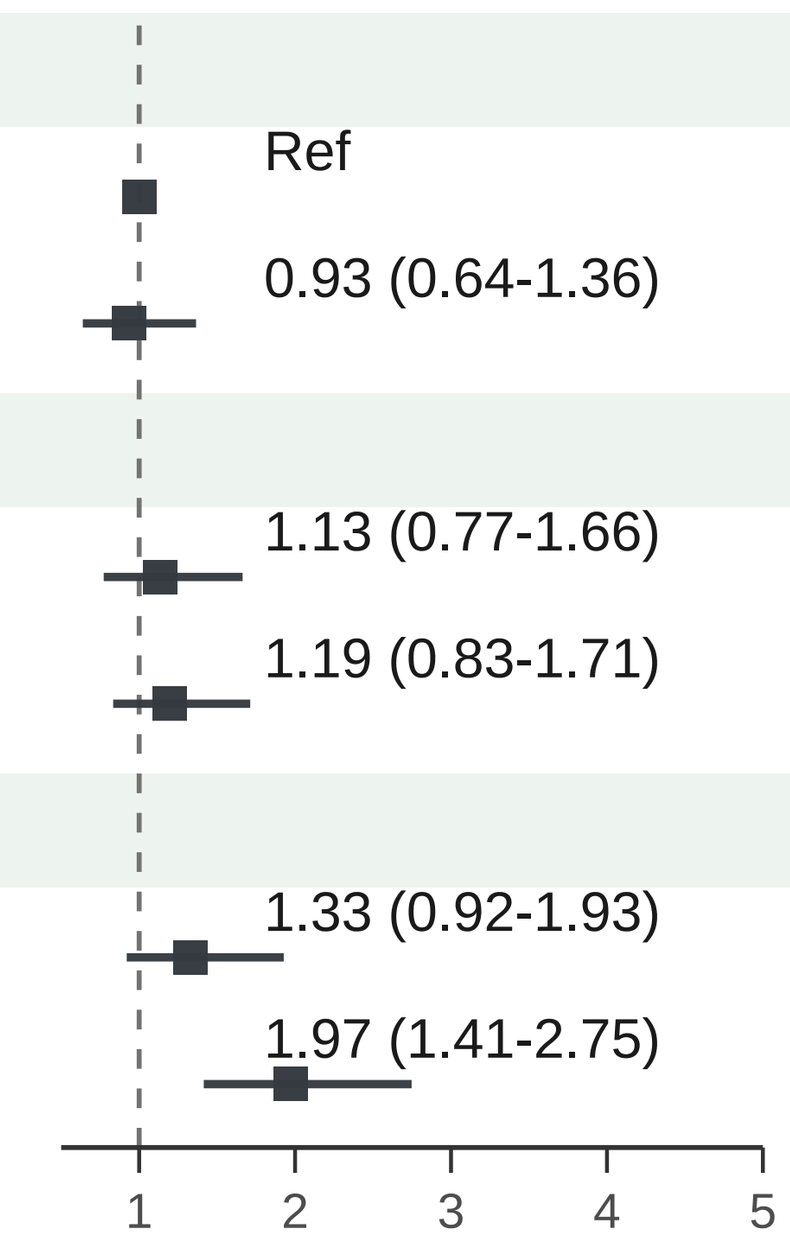

E Kidney cancer × CDAI

Interaction P = 0.990
High genetic risk: diet HR per 1-SD = 0.88 (0.75-1.04); P = 0.142

Exposure	Outcome	Case	Control	HR (95% CI)	P
**Low Genetic Risk**					
High CDAI score	Kidney cancer	33	12769	Ref	
Low CDAI score	Kidney cancer	35	12831	0.93 (0.57-1.51)	0.758
**Intermediate Genetic Risk**					
High CDAI score	Kidney cancer	26	12840	0.78 (0.47-1.30)	0.343
Low CDAI score	Kidney cancer	55	12793	1.43 (0.92-2.23)	0.113
**High Genetic Risk**					
High CDAI score	Kidney cancer	54	12859	1.58 (1.03-2.44)	0.038
Low CDAI score	Kidney cancer	76	12791	2.02 (1.33-3.07)	0.001

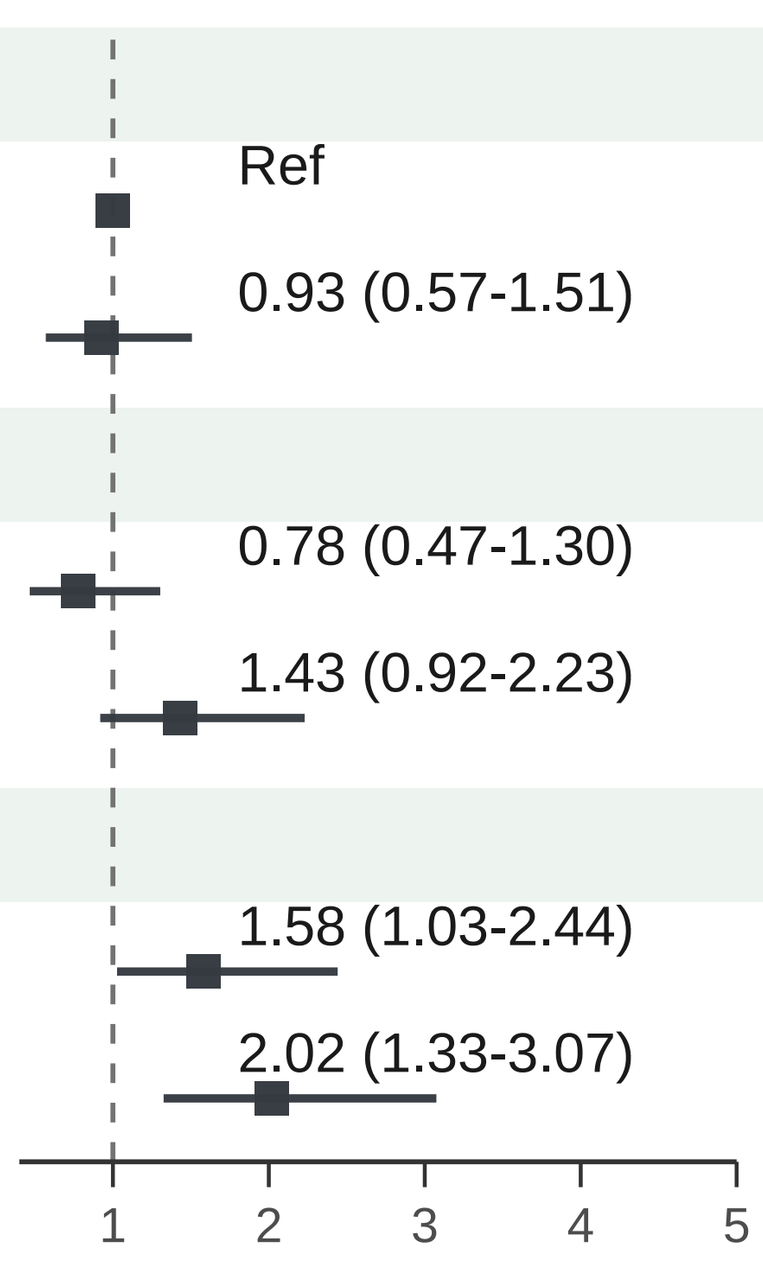

F Kidney cancer × DASH

Interaction P = 0.013
High genetic risk: diet HR per 1-SD = 0.78 (0.67-0.91); P = 0.002

Exposure	Outcome	Case	Control	HR (95% CI)	P
**Low Genetic Risk**					
High DASH score	Kidney cancer	41	14752	Ref	
Low DASH score	Kidney cancer	38	14815	0.98 (0.63-1.53)	0.936
**Intermediate Genetic Risk**					
High DASH score	Kidney cancer	45	14733	1.10 (0.72-1.68)	0.659
Low DASH score	Kidney cancer	51	14697	1.30 (0.86-1.98)	0.213
**High Genetic Risk**					
High DASH score	Kidney cancer	52	14883	1.25 (0.83-1.88)	0.292
Low DASH score	Kidney cancer	90	14609	2.35 (1.61-3.41)	<0.001

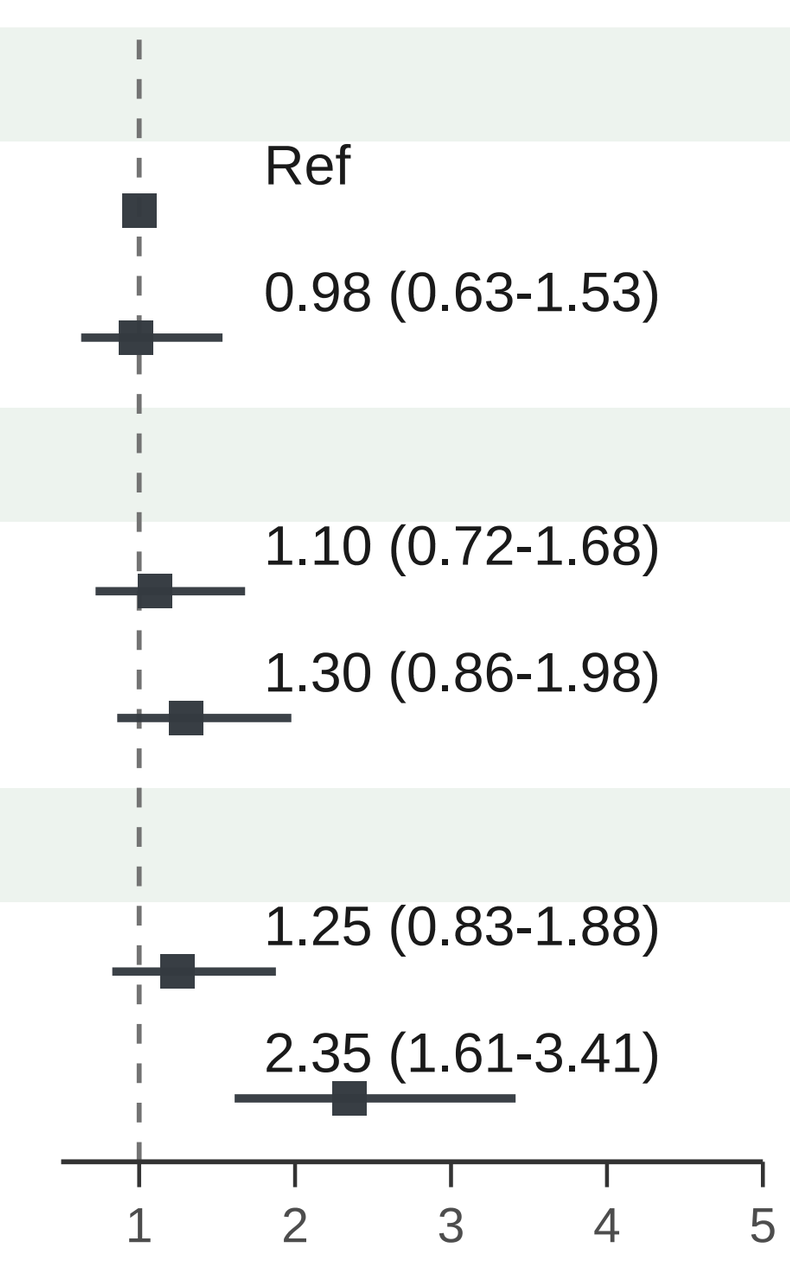

G Kidney cancer × Reversed E-DII

Interaction P = 0.956
High genetic risk: diet HR per 1-SD = 0.80 (0.66-0.97); P = 0.025

Exposure	Outcome	Case	Control	HR (95% CI)	P
**Low Genetic Risk**					
High Reversed E-DII score	Kidney cancer	33	12872	Ref	
Low Reversed E-DII score	Kidney cancer	32	12908	0.88 (0.53-1.46)	0.631
**Intermediate Genetic Risk**					
High Reversed E-DII score	Kidney cancer	31	12801	0.95 (0.58-1.55)	0.837
Low Reversed E-DII score	Kidney cancer	55	12781	1.48 (0.94-2.33)	0.090
**High Genetic Risk**					
High Reversed E-DII score	Kidney cancer	45	12799	1.35 (0.86-2.11)	0.193
Low Reversed E-DII score	Kidney cancer	65	12740	1.79 (1.16-2.79)	0.009

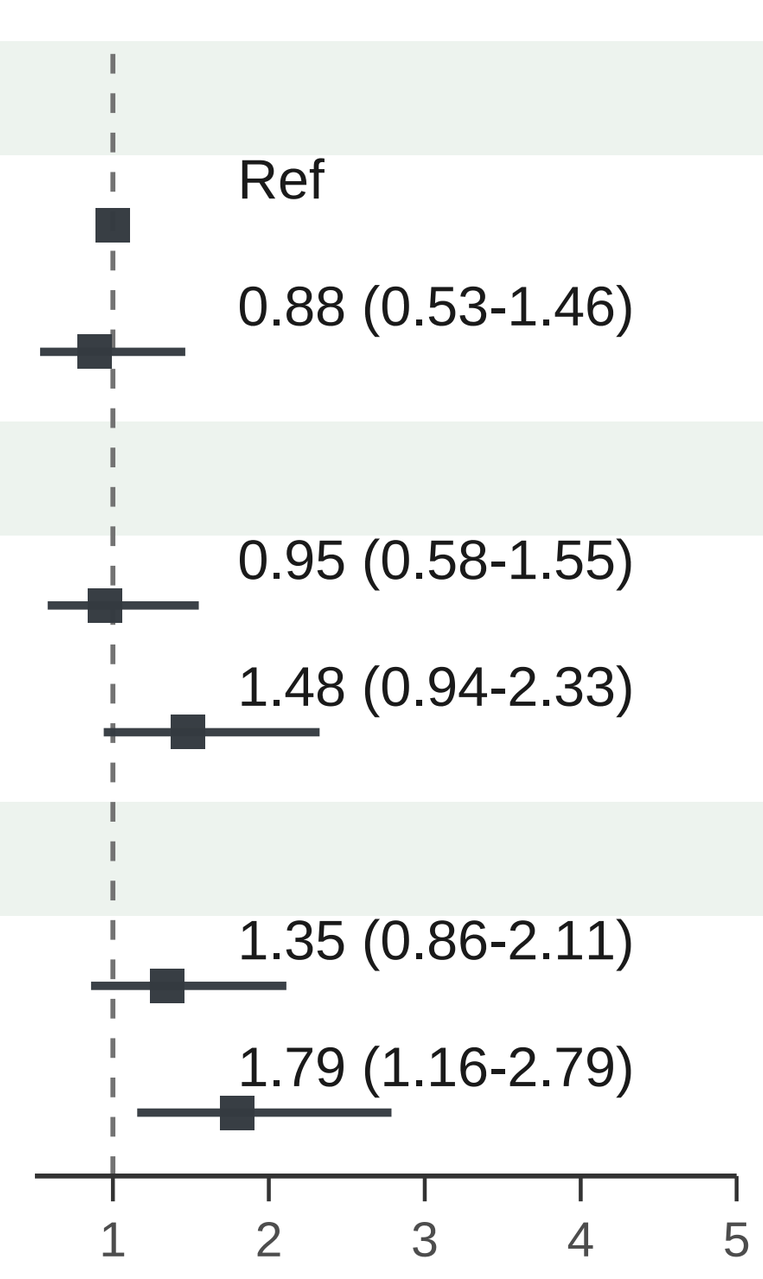

H Lung cancer × AHEI

Interaction P = 0.703
High genetic risk: diet HR per 1-SD = 0.88 (0.78-0.98); P = 0.022

Exposure	Outcome	Case	Control	HR (95% CI)	P
**Low Genetic Risk**					
High AHEI score	Lung cancer	62	12919	Ref	
Low AHEI score	Lung cancer	76	12750	1.10 (0.78-1.55)	0.577
**Intermediate Genetic Risk**					
High AHEI score	Lung cancer	75	12742	1.23 (0.88-1.72)	0.233
Low AHEI score	Lung cancer	120	12613	1.72 (1.26-2.35)	<0.001
**High Genetic Risk**					
High AHEI score	Lung cancer	95	12688	1.57 (1.14-2.16)	0.006
Low AHEI score	Lung cancer	152	12870	2.15 (1.59-2.90)	<0.001

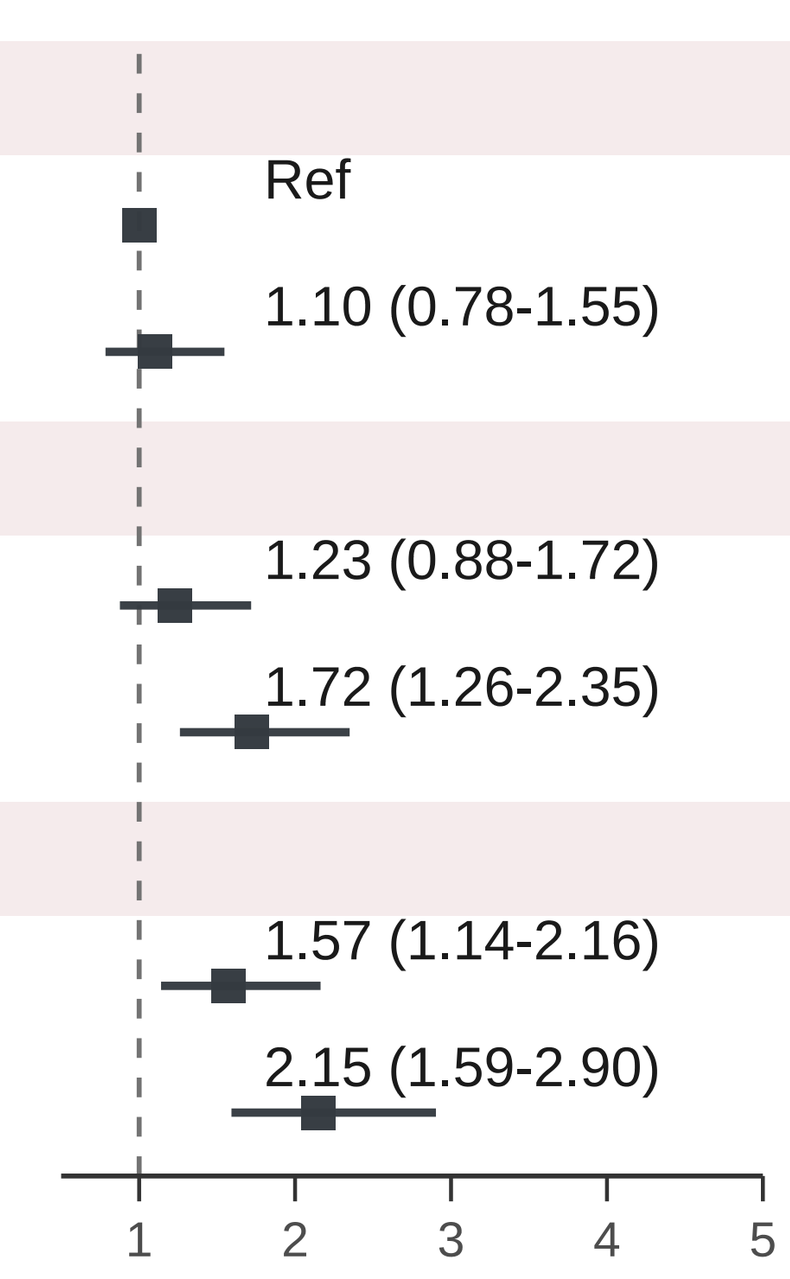

I Lung cancer × aMED

Interaction P = 0.632
High genetic risk: diet HR per 1-SD = 0.93 (0.84-1.04); P = 0.213

Exposure	Outcome	Case	Control	HR (95% CI)	P
**Low Genetic Risk**					
High aMED score	Lung cancer	93	18099	Ref	
Low aMED score	Lung cancer	112	20277	0.98 (0.74-1.29)	0.870
**Intermediate Genetic Risk**					
High aMED score	Lung cancer	115	17998	1.22 (0.93-1.61)	0.146
Low aMED score	Lung cancer	193	20274	1.67 (1.30-2.14)	<0.001
**High Genetic Risk**					
High aMED score	Lung cancer	157	17890	1.71 (1.32-2.21)	<0.001
Low aMED score	Lung cancer	213	20320	1.83 (1.43-2.34)	<0.001

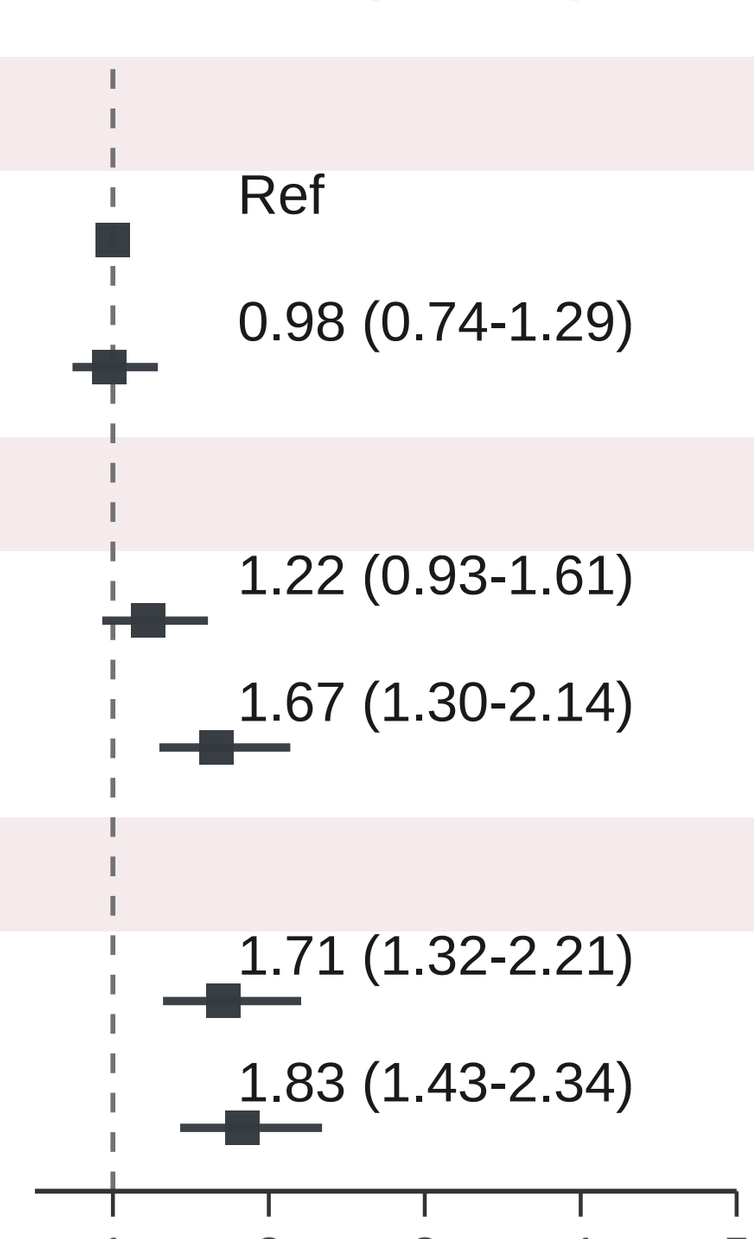

J Lung cancer × Reversed E-DII

Interaction P = 0.289
High genetic risk: diet HR per 1-SD = 0.90 (0.79-1.02); P = 0.098

Exposure	Outcome	Case	Control	HR (95% CI)	P
**Low Genetic Risk**					
High Reversed E-DII score	Lung cancer	68	12890	Ref	
Low Reversed E-DII score	Lung cancer	75	12725	1.04 (0.74-1.46)	0.823
**Intermediate Genetic Risk**					
High Reversed E-DII score	Lung cancer	96	12752	1.39 (1.02-1.90)	0.037
Low Reversed E-DII score	Lung cancer	119	12765	1.65 (1.21-2.25)	0.002
**High Genetic Risk**					
High Reversed E-DII score	Lung cancer	102	12673	1.50 (1.11-2.04)	0.009
Low Reversed E-DII score	Lung cancer	145	12752	2.00 (1.48-2.71)	<0.001

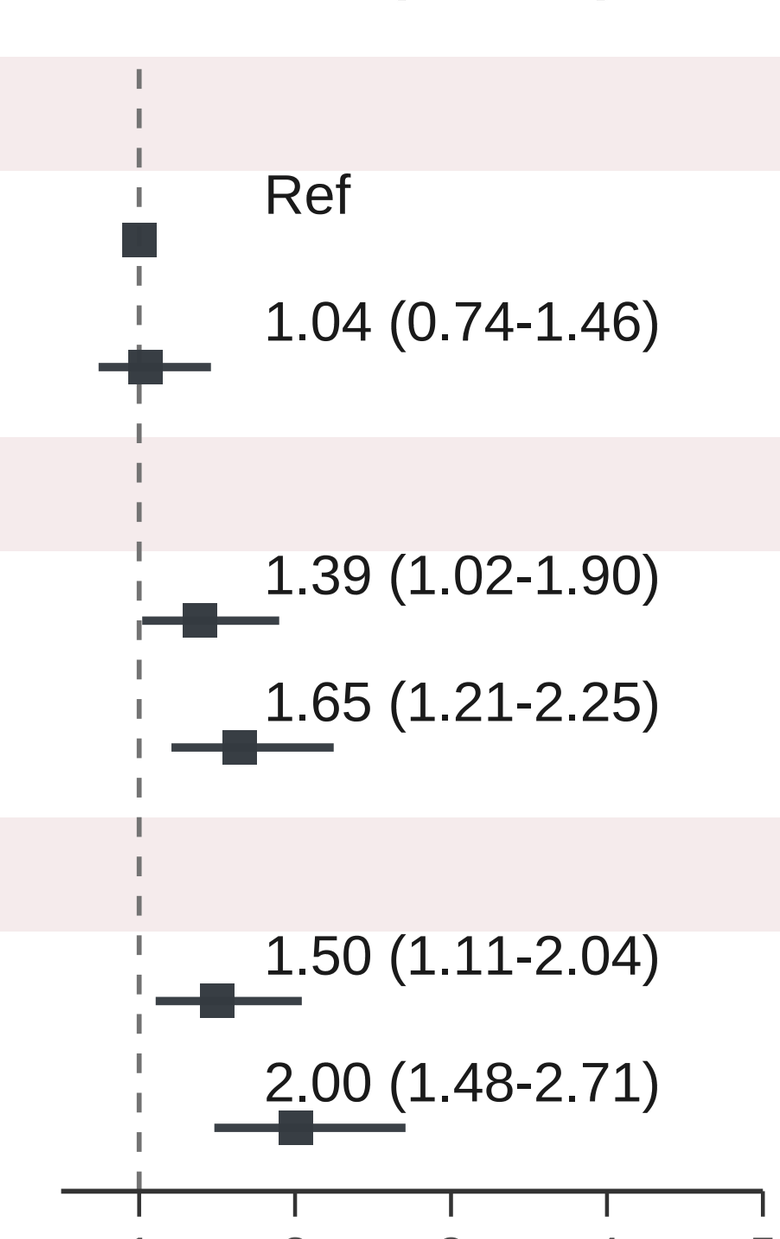

Model 3 adjusted.
Reference group: low genetic risk + healthier diet group.
Diet score groups were defined by tertiles of standardized dietary scores: Low = bottom one-third, High = top one-third; the middle one-third was not shown in the joint-group forest plot.
PRS groups were defined by tertiles: Low, Intermediate, and High genetic risk.
Interaction was tested using continuous PRS × continuous standardized dietary score. Reversed E-DII indicates E-DII after reverse standardization; higher values represent lower dietary inflammatory potential.
