# Supplementary material for: Comparative associations of six predefined dietary patterns with site-specific cancer risk in the UK Biobank
Source: Front Nutr. 2026 Oct 8;13:1976233. doi: 10.3389/fnut.2026.1976233 (PMC13686994; doi:10.3389/fnut.2026.1976233)
Supplement: Supplementary file 2 [file Image_2.PDF]

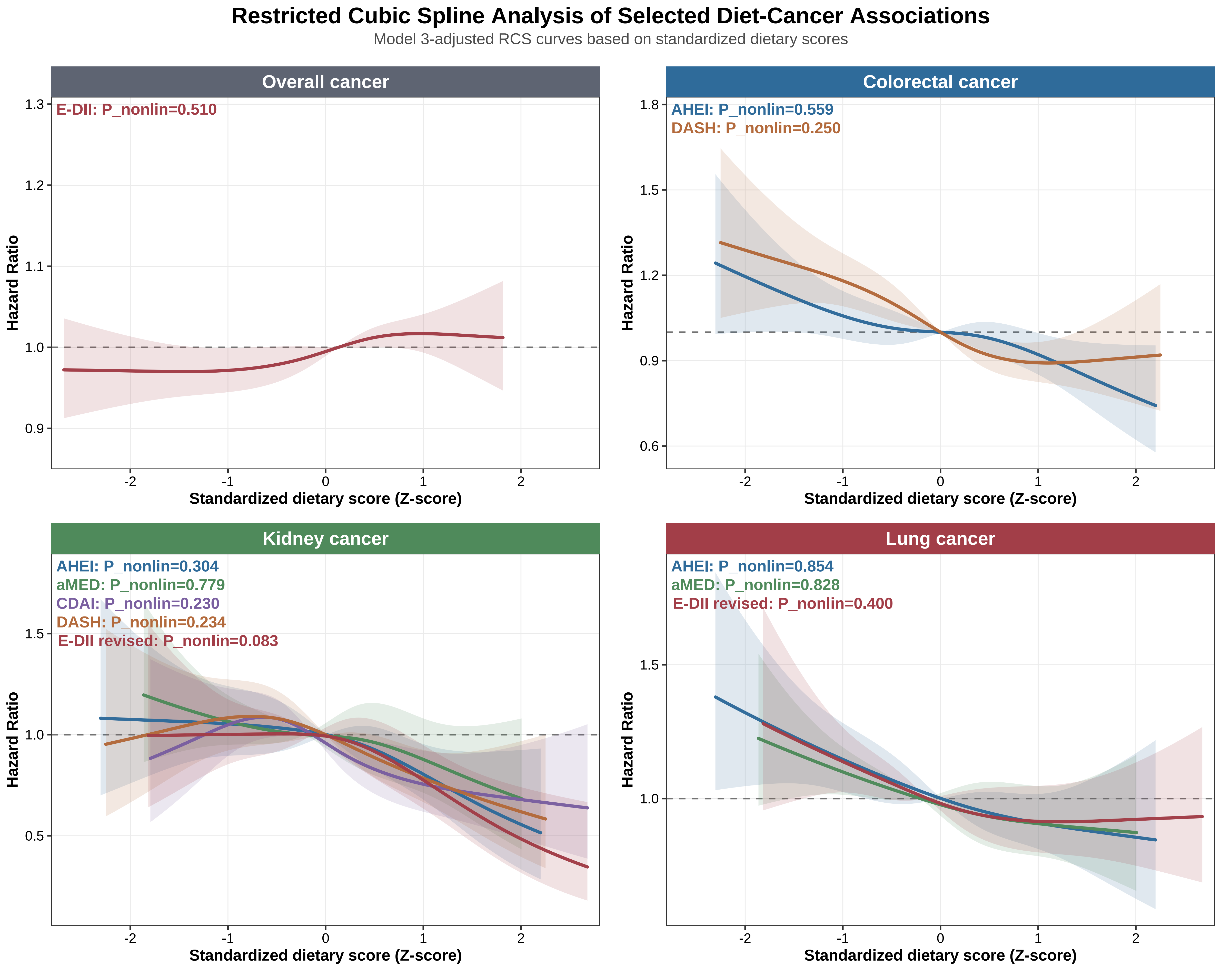

P_nonlin indicates the P value for nonlinearity. * P<0.05, ** P<0.01, *** P<0.001. E-DII reversed indicates reverse-standardized E-DII; higher values represent a less pro-inflammatory dietary pattern.
