# Supplementary material for: Comparative associations of six predefined dietary patterns with site-specific cancer risk in the UK Biobank
Source: Front Nutr. 2026 Oct 8;13:1976233. doi: 10.3389/fnut.2026.1976233 (PMC13686994; doi:10.3389/fnut.2026.1976233)
Supplement: Supplementary file 3 [file Image_3.PDF]

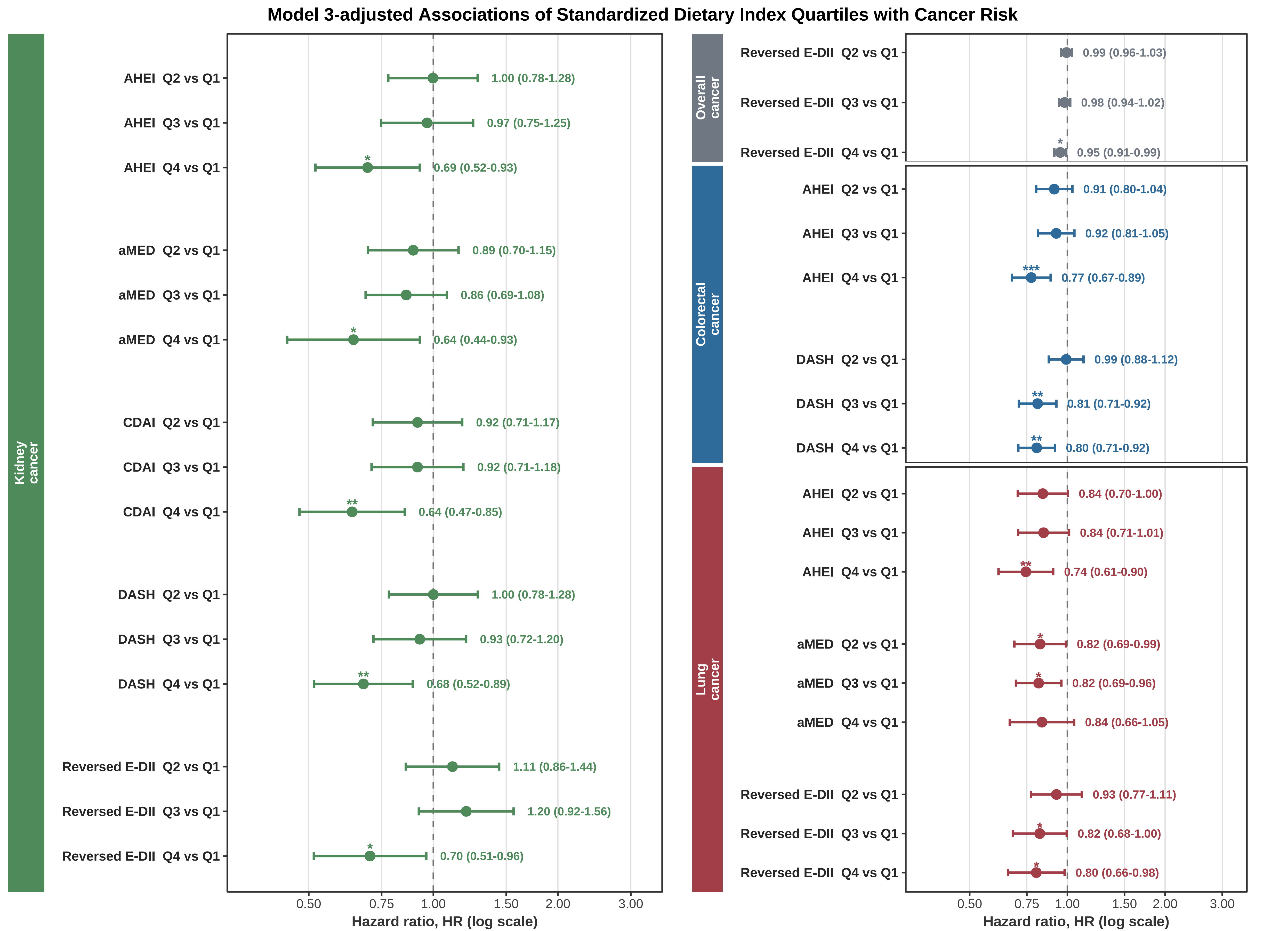

Model 3 adjusted. Points show HRs with 95% CIs; values at right are HR (95% CI). * P<0.05, ** P<0.01, *** P<0.001. Reference setting: all selected dietary indices use Q1, the least healthy quartile.
