# Supplementary material for: Comparative associations of six predefined dietary patterns with site-specific cancer risk in the UK Biobank
Source: Front Nutr. 2026 Oct 8;13:1976233. doi: 10.3389/fnut.2026.1976233 (PMC13686994; doi:10.3389/fnut.2026.1976233)
Supplement: Supplementary file 4 [file Supplementary_file_1.DOCX]

**Supplementary Material**

**Comparative associations of six predefined dietary patterns with site-specific cancer risk in the UK Biobank**

**Supplementary Methods: Extended Analysis – Joint Analysis of Dietary Patterns and Genetic Risk**

**Supplementary Tables:**

**Table S1** Extraction of 24-hour dietary intake variables and construction of dietary indices

**Table S2** Summary of covariate and outcomes definitions

**Table S3** Summary of cancer diagnoses and event threshold

**Table S4** Primary analysis—Cox regression of 6 dietary indices with 15 cancers—screening

**Table S5** Primary analysis—1y3y5y lag sensitivity analysis for significant pairs—validation

**Table S6** Exploratory analysis—quartile-based dose–response of stable diet–cancer pairs

**Table S7** Joint analysis of dietary patterns and PRS strata for stable diet–cancer pairs

**Table S8** Exploratory analysis—dietary index comparison within cancer—bootstrap pairwise comparison

**Table S9** Exploratory analysis—dietary index comparison by PRS strata—bootstrap pairwise comparison

**Table S10** Comparison of present study with previous evidence

**Supplementary Figures:**

**Figure S1** Interaction analyses of genetic risk stratification and dietary patterns in colorectal cancer, lung cancer, and kidney cancer

**Figure S2** Exploratory analysis—dose–response relationships of stable diet–cancer pairs using restricted cubic splines

**Figure S3** Exploratory analysis—quartile-based dose–response and rank comparison of stable diet–cancer pairs

**Supplementary Methods: Extended Analysis – Joint Analysis of Dietary Patterns and Genetic Risk**

To assess the value of dietary patterns for risk stratification under genetic susceptibility, we further conducted joint analyses of dietary patterns and PRS for dietary pattern–cancer pairs that showed stable associations in the preceding sensitivity analyses. Publicly available single-nucleotide polymorphism (SNP) scoring files for the target cancers were obtained from the Polygenic Score (PGS) Catalog. Within the UK Biobank Research Analysis Platform (UKB-RAP), individual SNP-weighted scores were calculated separately by chromosome using embedded PLINK in the Swiss Army Knife application, with SNP effect sizes taken directly from the original publications as beta coefficients or log odds ratios. All PRSs were aligned and calculated based on the Genome Reference Consortium Human Build 37 (GRCh37; hg19) reference genome. Chromosome-specific scores were then summed by individual identifier to derive the total PRS. After standardization, each cancer-specific PRS was categorized into low, intermediate, and high genetic risk groups by tertiles. Dietary pattern scores were similarly divided into tertiles, although only the lower and upper tertiles (Low vs High) were retained for joint visualization. A six-category joint variable combining genetic risk and extreme dietary pattern groups was then constructed. Within the fully adjusted Model 3 framework, hazard ratios (HRs) and 95% confidence intervals (CIs) were estimated for each joint category, using the low genetic risk group with the healthy dietary pattern as the reference. To examine interaction between dietary patterns and genetic susceptibility, continuous PRS, continuous dietary pattern scores, and their product term were simultaneously entered into the Cox model, and a likelihood ratio test comparing nested models with and without the interaction term was used to derive the P for interaction. In addition, separate Cox models were fitted within the high genetic risk stratum to assess the associations between continuous dietary pattern scores and cancer risk.
